# Human-centred design of a new microneedle-based hormonal contraceptive delivery system

**DOI:** 10.12688/gatesopenres.13233.1

**Published:** 2021-06-17

**Authors:** Benedetta Gualeni, Louise Hughes, Isabelle Stauber, Louise Ackers, Angela Gorman, Dorothy Gashuga, Nettie Dzabala, Sion A. Coulman, James C. Birchall

**Affiliations:** 1 School of Pharmacy and Pharmaceutical Sciences, Cardiff University, Redwood Building, King Edward VII Avenue, Cardiff, CF10 3NB, UK; 2 Maddison Limited, Walnut Tree Yard, Lower Street, Fittleworth, RH20 1JE, UK; 3 School of Health and Society, University of Salford, Allerton Building, Salford, M6 6PU, UK; 4 Knowledge for Change, Plot 39 Saaka Road, Kagote, Fort Portal, P.O. Box 392, Uganda; 5 Life for African Mothers, Suite 18, Big Yellow Storage, Cardiff, CF10 5DL, UK; 6 College of Medicine, University of Malawi, Mahatma Gandhi Road, Blantyre, Malawi

**Keywords:** Human-centred design (HCD), Microneedles (MNs), Hormonal contraceptive, User studies, Family Planning, Low- and middle-income countries (LMICs), Medical device development.

## Abstract

**Background:** It is estimated that 225 million women worldwide have an unmet need for family planning, and more than half live in low- and middle-income countries. Increasing the choice of contraceptive methods available can reduce this unmet need. Microneedle drug delivery systems represent a new technology for minimally invasive self-administration of contraceptives. We explored stakeholders’ views on different aspects of a proposed microneedle-based hormonal contraceptive delivery system. The feedback was used to iteratively develop this delivery system.

**Methods:** Focus group discussions and semi-structured interviews were conducted with potential stakeholders (women and trans males of childbearing age, their partners, and health professionals and organisations that provide family planning advice and contraception services) in Uganda, The Gambia, Malawi, and the UK, exploring concept acceptability and gathering feedback on different aspects of design and usability of the proposed delivery system.

**Results:** Participants viewed the concept of a new, microneedle-based contraceptive favourably. In Uganda, participants were presented with 7 different prototype applicators and identified desirable features of a preferred delivery device; their input reducing the number of prototypes that were subsequently evaluated by stakeholders in The Gambia and the UK. Participants in these countries helped to identify and/or confirm the most desirable characteristics of the applicator, resulting in design consolidation into a refined concept applicator. The final, optimised applicator prototype was validated during user research in Malawi. This human-centred design approach was also used to iteratively develop an information leaflet for the device. During these user studies, other preferred aspects of a contraceptive delivery system were also evaluated, such as anatomical site of application, duration of action, and return to fertility.

**Conclusions:** A new microneedle-based contraceptive delivery system was iteratively developed using a human-centred design approach and was favourably received by potential stakeholders. The product is now being refined for testing in pre-clinical studies.

## Introduction

The United Nations (UN) Sustainable Development Goals for 2030 recognise the importance of Family Planning (FP) as a key component of global good health and wellbeing (Goal 3.7) ^1^. In addition to providing decision-making autonomy on whether, when and how many children to have, numerous studies have demonstrated that increasing access to FP can reduce maternal and infant mortality, decrease unsafe abortions, increase educational prospects, and reduce poverty ^2– 10^.

Despite a worldwide increase in contraception use, it is estimated that currently 225 million women worldwide have an unmet need for FP (to limit or space births). More than half of the women with this unmet need live in low- and middle-income countries (LMICs), including countries in Sub-Saharan Africa (SSA) ^11^. A wide range of factors influence contraceptive use including, but not limited to, socio-cultural norms, socio-economic status, education and occupational status, proximity to FP clinics, knowledge and understanding of methods, availability of educational tools, fear of side effects, gender power imbalances, provider’s skill and personal bias, and ability to discuss FP with partners, friends, and healthcare providers ^12– 16^. Enhanced education and improved access to FP is paramount to satisfy the unmet FP needs of individuals in LMICs ^17, 18^. Increasing the number and variety of methods of contraception available to potential end-users has also been proposed as a strategy to help reduce the unmet need for FP in these countries ^12, 19^. A new minimally invasive microneedle-based hormonal contraceptive delivery system that requires minimal training and is easy-to-use is therefore potentially an attractive proposition ^20^.

Microneedles (MNs) are medical devices containing microscopic needle shaped projections that can be applied to the skin to deliver a wide range of therapeutics in a minimally invasive, painless, and safe manner ^21– 24^. Biodegradable polymer MNs are able to facilitate controlled release of the therapeutic cargo ^25^ and therefore could be exploited for the release of hormonal contraceptives over extended periods of time (≥1month) ^26– 29^.

In this study, an innovative MN-based, progestin-only hormonal contraceptive delivery system with the potential for self-administration was explored with prospective stakeholders. A human-centred design (HCD) approach was used to directly inform specific design features of the system and develop prototype products. HCD, which originated in the computer science and artificial intelligence fields ^30^, is increasingly being used to design novel solutions for complex problems in global health ^31^, including reproductive health ^32– 34^. This highly multidisciplinary method incorporates the voice of end users throughout all stages of iterative product development. In this study, end users and stakeholders were recruited and engaged to inform the aesthetics and usability of a proposed new MN-based delivery system, and to iteratively develop a user-friendly instruction leaflet. During the study, participants were also encouraged to share their thoughts on the proposed mode of administration, mode of access, duration of contraceptive effect, and time to return to fertility after discontinuation of the contraceptive. Feedback facilitated the development of a user-informed delivery system with desirable features and iterative optimisation of prototypes, resulting in a validated optimised product that is currently being tested in pre-clinical studies.

## Methods

### Concept development

**Figure 1.**
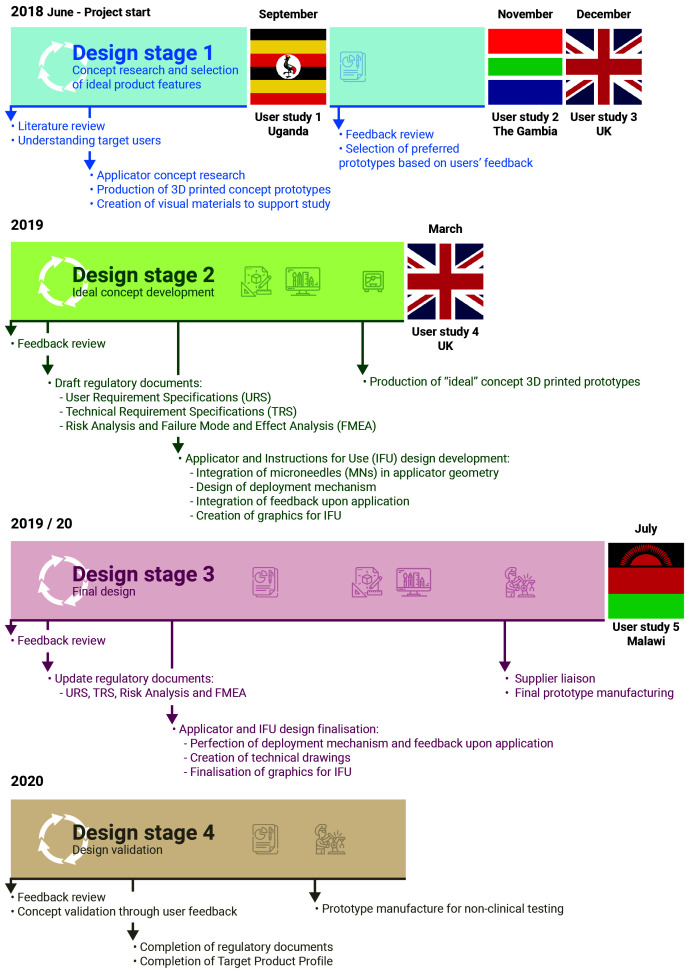
Project timeline. Following the HCD approach schematised in Figure 2, the project started with concept research based around the published literature on the unmet need for contraception in LMICs. Multiple prototypes covering a wide range of desirable features for a contraceptive product were produced and evaluated by potential stakeholders in LMICs. Participant feedback helped refine the product according to stakeholders’ preferences and consolidate the final product design. this was then validated through a final users’ evaluation prior to pre-clinical testing.

**Figure 2.**
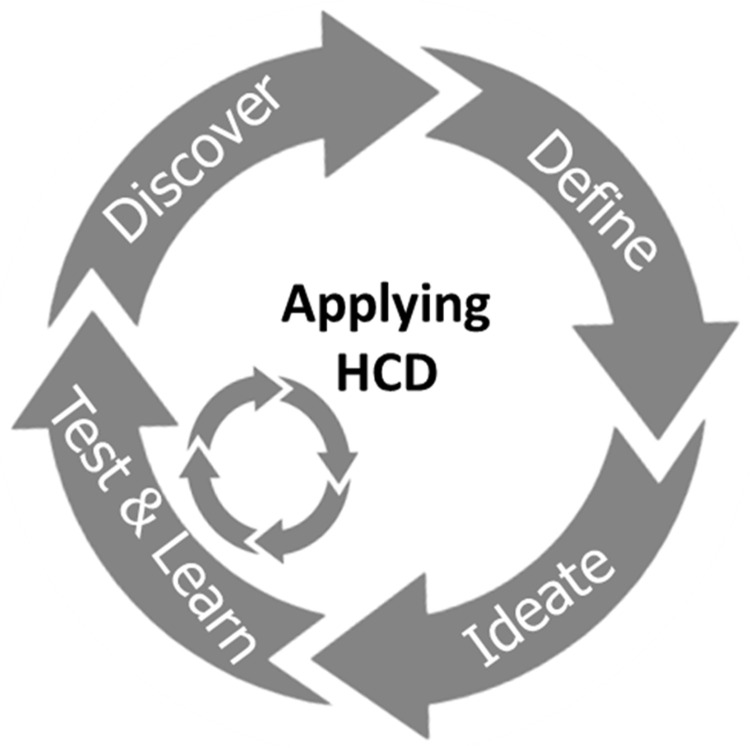
Diagram illustrating the different steps of a Human Centred Design study. After defining the area of innovation, the target end users and the context of use are considered to ideate possible solutions for the unmet need that is being addressed. The proposed solutions are tested by potential end users and their feedback is incorporated into a refined design. Multiple iterations of this process lead to the development of the final product.

The concept of a new MN-based progestin-only hormonal contraceptive delivered by an applicator was developed by the team (Design Stage 1, Figure 1) after reviewing the available literature and discussing the expectations of stakeholders regarding novel contraception solutions with experts in the field of FP in LMICs. A HCD approach ( Figure 2) was used to integrate the views of potential end users and other key stakeholders at all stages of product development. A detailed timeline of the performed activities is provided in Figure 1.

A total of 31 FGDs and 15 SSIs were conducted with participants in Uganda (September 2018 – Stage 1), The Gambia (November 2018 – Stage 1), The UK (December 2018 – Stage 1; March 2019 – Stage 2), and Malawi (July 2020 – Stage 3) to explore participants’ feedback and preferences regarding different aspects of the applicator design and appearance of the instruction leaflet. During FGDs/SSIs, participants views were also explored regarding the proposed anatomical sites of application, scenarios for contraceptive provision, duration of action, acceptable time to return to fertility after discontinuation, and any other identified benefits and/or concerns; these are described in more detail in the subsequent text.

### Study design

 A pragmatic, qualitative approach was developed due to the exploratory nature of the research aims. Semi-structured interviews (SSIs) and focus group discussions (FGDs) were used, with the choice of method dictated by local needs, participant preference, and ability to arrange homogeneous groups. Where no preference was expressed by the participants and there were no logistical issues, FGDs were the preferred method due to the dynamic nature of discussion amongst participants leading to increased opportunity to understand their views ^35^.

Illustrated scenarios and prototype delivery systems were used during FGDs and SSIs. This allowed participants to visualise the concept, interact with the prototypes and express their preferences. Pre-interview questionnaires were also used to obtain demographic information and to understand participants’ views and experiences of contraception.

### Ethical considerations

Ethical approvals for the studies were sought and obtained from Cardiff University School of Pharmacy and Pharmaceutical Sciences Research Ethics Committee (UK) (SREC references: 1718–29 and 1920–17), University of Salford Research Ethics Committee (UK) (HSR1617-129), Mountains of the Moon University Research Ethics Panel (MMU/DPGSR/061218), the Gambia Government/MRCG Joint Ethics Committee (R018030) and the College of Medicine Research Ethics Committee (Malawi) (COMREC reference: P.01/19/2580).

Participants provided written informed consent prior to data collection; a detailed information sheet was provided, and potential participants were able to ask questions before deciding whether to participate. Due to the potentially sensitive nature of the topic, participants were reminded of the confidentiality of discussions at the beginning of the exercise. SSIs and FGDs were held in locations convenient to the participants, and where they could not be overheard by others. All interview transcripts were anonymised. Recordings and transcripts were retained on a password protected folder only accessible to the research team and all paperwork (consent forms and screening questionnaires) held in a locked filing cabinet.

### Development of research materials

A demographic questionnaire was developed to obtain brief background information about the participants. This was based on published research detailing those factors which might influence views and preferences of FP, including age, religion, family status, education and occupational status, previous and current experience with contraception ^12– 17^, and allowed the research team to ensure a range of participants with different background characteristics were included in the SSIs/FGDs.

A topic guide for the FGDs/SSIs was designed to explore the participants’ general views around current contraceptive options, to provide context, before moving on to discuss the proposed new method in more detail. This second part of the topic guide included broad questions about the concept, following a brief introductory explanation, and the participants were then shown a range of prototype delivery systems and associated instructions. Questions and prompts related to the participant’s preferences around the aesthetics, functionality, and usability of these prototypes, as well as suggestions for improvements. Questions were based on published research literature, discussions within the team, and specific technical features that required prospective stakeholder input. Advice from the Population Council (an international, non-profit, non-governmental organisation specialising in reproductive health), local SSA researchers, local SSA District Health Officers and contacts who undertake charity, research and health work in the countries concerned was crucial for developing the topic guide described above, in order to assure its local socio-cultural acceptability. In addition, two pilot FGDs (one with potential users, and one with potential providers) were used to test the topic guide; no changes were required. Feedback from the Bill & Melinda Gates Foundation (who provided funding for this research) also informed development of materials. All materials used in the study can be found as extended data ^36^.

Illustrated scenarios and prototype 3D delivery systems were produced by Maddison Ltd. (a specialist product design consultancy), offering a range of options regarding potential use environment and design features. These enabled participants to imagine using the product, handle prototype models, and give more specific feedback about design, functionality, and usability.

Different versions of user instructions were also prepared, ranging from very detailed text instructions to simplified coloured visual images, and feedback on the clarity and messaging in these resources was sought during FGDs and SSIs.

### Sampling and recruitment

**Figure 3.**
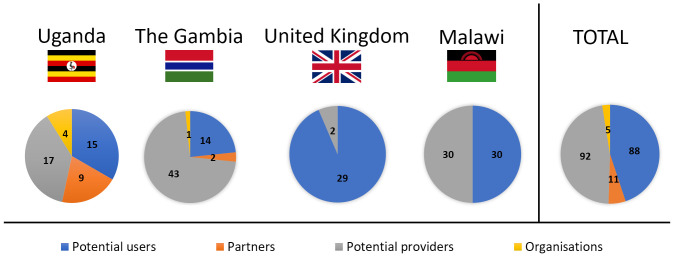
Participants in the study grouped by country. Four different groups were included in focus group discussions and semi-structured interviews: potential users (blue), partners of potential users (orange), potential providers (grey) and organisations that provide family planning (yellow).

Feedback from four distinct groups was sought: potential users of the proposed contraceptive solution (Uganda, The Gambia, Malawi, United Kingdom), men whose partners are potential future users of such a product (Uganda, The Gambia), health professionals who are potential suppliers of the proposed device (Uganda, The Gambia, Malawi, United Kingdom), and organisations that provide FP advice and contraception in SSA (Uganda, The Gambia) ( Figure 3). There were no sample size calculations, rather data collection continued until all opportunities had been exhausted within the limits of the data collection period, e.g. the duration of the field visit (Uganda, The Gambia, UK). In Malawi a target of 30 users and 30 providers was identified as being realistic to provide sufficient feedback within the research timescales, based on experience in earlier phases. Each group is considered in turn below.

Eligible potential users (n=88) were classed as women or trans males of childbearing age (nominally aged 18–45), regardless of prior or current contraceptive use or non-use. Recruitment was predominantly through convenience and purposive sampling. Local gatekeepers used their knowledge to purposively sample and recruit (face-to-face or via telephone) via different contacts and settings to facilitate a diverse demographic (age, education, living environment, marital status, family status, use of contraception, religion) that would encompass different views and preferences ^12– 16, 37^. In addition, posters were placed in community venues to allow individuals to self-select (UK only).

Partners of potential users (n=11) were loosely defined as men whose partners may consider using contraception. They were convenience-sampled opportunistically using local links, and invited to participate, since prior research has shown that in some settings male partners’ views can play a significant role in the use (or non-use) of contraception ^38– 40^.

Potential providers (n=92) were defined as health professionals of any type, whose role involves the provision of contraceptive advice and / or methods. They were recruited through clinics, workplaces, and local contacts, using purposive sampling to identify providers with a range of roles (medics, nurses, midwives, interns, and healthcare students) and experience in different work environments (rural or urban). Other characteristics (age, religion, personal experience with contraception) were also identified where possible, to check for the further diversity of the sample.

Organisational stakeholders (n=5) were identified by team members and local links using convenience sampling. These were defined as individuals working for non-governmental organisations, which play a role in the provision of contraception in the relevant countries. Typically, an introduction was made initially by a local contact and then the interview set up directly by the researchers. Interviews with these key informants focused on their views on the proposed delivery system in terms of acceptability within the country.

### Data collection process

A questionnaire (as described above) was used with all groups, except organisational stakeholders, to identify relevant background/demographic data. This was provided alongside the participant information sheet and requested to be returned, completed, to the researchers before or during the interview or FGD if they consented to participate. A semi-structured topic guide (as discussed above) was used to guide the SSIs/FGDs performed at Design Stage 1, 2, and 3 which were audio-recorded, with consent. Where consent was not given for audio-recording, detailed written notes of the conversation were taken with the consent of the participant.

Interviews and FGDs in the UK, The Gambia and Uganda were undertaken by members of the research team or their students with experience and/or training in data collection methods (n=6, JB, BG, LH, IS plus 2 MPharm students). (FGDs/SSIs conducted in English, or through pragmatic use of a local, neutral translator). Local, trained, researchers collected the data in Malawi (FGDs conducted in Chichewa, translated for data analysis, and back-translated to ensure the accuracy of transcription). Typically, FGDs involved two researchers and SSIs one researcher, with the exception of SSIs with organisations. Female researchers were used to collect data from potential users in case any participants would find it difficult or inhibitory to talk to a male researcher about the topic. Conversely, where possible male researchers collected data from partners. Researchers presented themselves as neutral members of the wider project team, with a focus on obtaining open and honest views on the new device to feed back to colleagues in the technical team. Participants were therefore encouraged to say what they genuinely thought about the idea without fear of offending anybody. 

The choice of whether data was collected via SSI or FGD depended on logistics and participant preference. Focus groups were ideally intended to have between 4–6 participants but a pragmatic approach was taken to ensure convenience for participants (e.g. pre-formed groups slightly larger than this were accepted). While groups involved only one type of stakeholder to ensure a degree of homogeneity, allocation to specific FGDs within each category was based predominantly on logistics. Each participant took part in just one SSI/FGD (I.e. the same individuals did not contribute to more than one SSI/FGD or stage of research). Locations were chosen based on convenience to the participants and their ability for the data collection to be carried out without being overhead or interrupted, for example a meeting room in a workplace or private space in a local community venue. Typically, interviews were anticipated to take 30 minutes and FGDs around 60–90 minutes. Although the time-limits on the field trips meant it was not necessarily possible to reach data saturation, the research team reflected on the experience and findings after each data collection point to ensure data was obtained on all of the key aspects as identified in the topic guide.

As noted above, SSIs/FGDs were audio-recorded with consent, and brief field notes were made where appropriate to supplement the recordings. During FGDs and SSIs with potential users, providers and organisations, dummy prototype MN applicators were used to obtain feedback on handling/ergonomics and aesthetics. This portion of the FGDs/SSIs was video recorded, with consent, to enable visual feedback on the handling of the delivery systems, with filming focused on the participants’ hands and not faces.

### Data analysis

Audio-recordings were transcribed, translated when necessary, anonymised, and thematically analysed. Data collection and analysis were iterative, with results from one stage feeding into the product design and enabling more focussed and detailed information to be collected in the next stage, as presented in Figure 1.

Coding and analysis were undertaken by three members of the research team with independent coding of the same data, followed by discussions, as an internal assurance check. At each stage, coding was carried out manually by reviewing and annotating transcripts line by line; decontextualization and recontextualization to group codes and development of themes was facilitated using MSWord®. A theoretical framework was not used: data were predominantly analysed inductively, with themes being derived from the data collected, although constant comparison was used to deductively review earlier SSIs/FGDs where a new theme arose from a later SSI or FGD. Deductive content analysis was used for the factual background information provided at the start of the SSI/FGD (e.g. providers’ roles and responsibilities).

 Logistically it was not possible to provide the transcripts or findings to the participants for comment, although all participants were provided with contact details for the research team should they wish to know more (none got in touch).

## Results

### Participants

 As presented in Figure 1, Stage 1 user studies were conducted in Uganda (User study 1), The Gambia (User study 2), and the United Kingdom (User study 3); Stage 2 user studies were conducted in the United Kingdom (User Study 4); Stage 3 user studies were conducted in Malawi (User study 5). An overview of the participants who took part in the user studies is presented in Figure 3. In User study 1, 15 potential users, 9 partners of potential users, 17 potential providers and 4 organisational stakeholders were confirmed as eligible and took part in the study. In User study 2, 14 potential users, 2 partners of potential users, 43 potential providers and 1 organisational stakeholder were confirmed as eligible and interviewed in SSIs/FGDs. In User study 3, 23 eligible potential users participated in the study. In User study 4, 6 potential users and 2 potential providers were confirmed as eligible and gave feedback on the concept refined during Stage 2. Finally, in User study 5, 30 potential users and 30 potential providers evaluated the final prototype designed during Stage 3.

**Figure 4.**
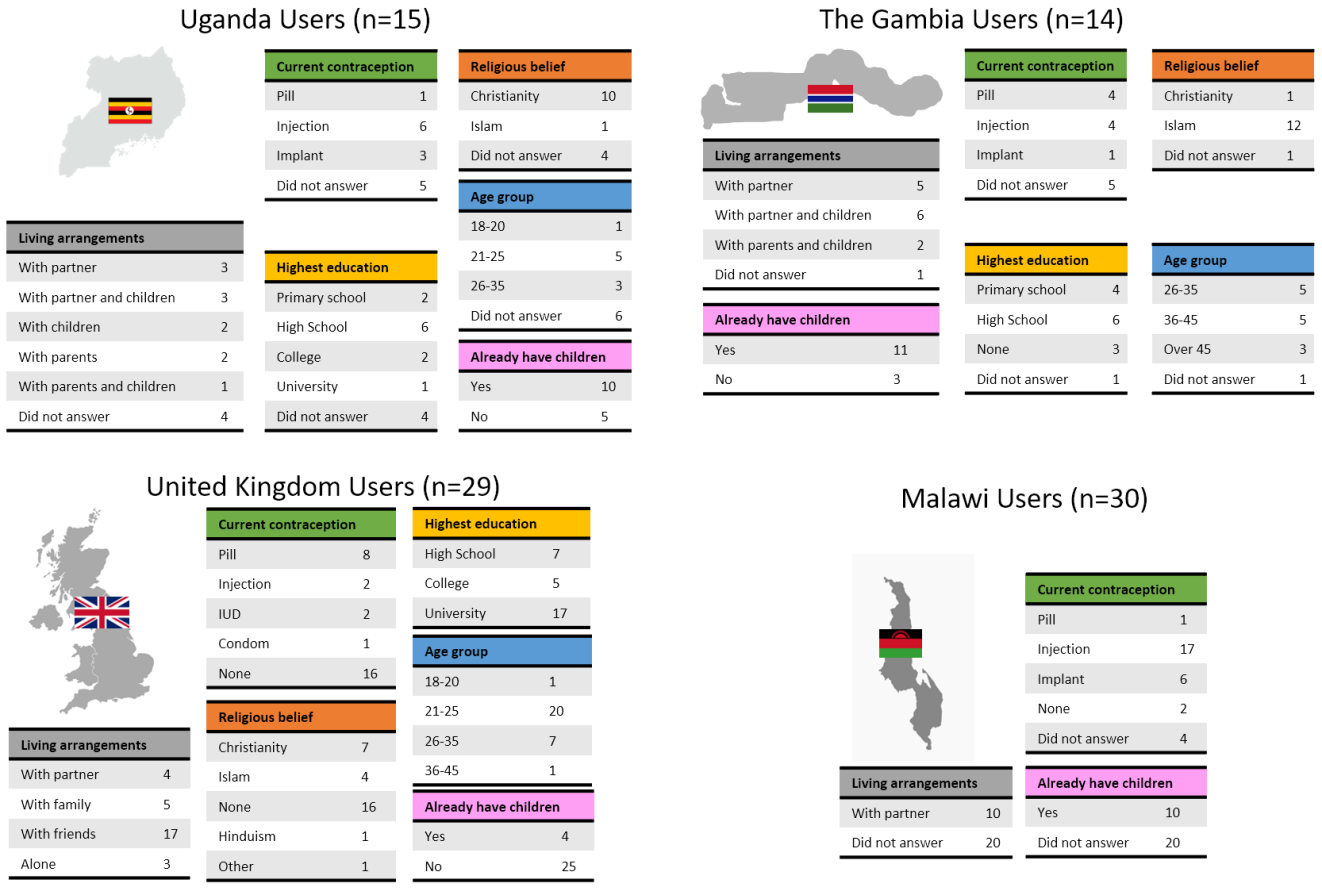
Demographic data of end users grouped by country. To receive comprehensive and diverse feedback, potential users of different age, education, religion, and at different stages of family planning were included in the study.

**Figure 5.**
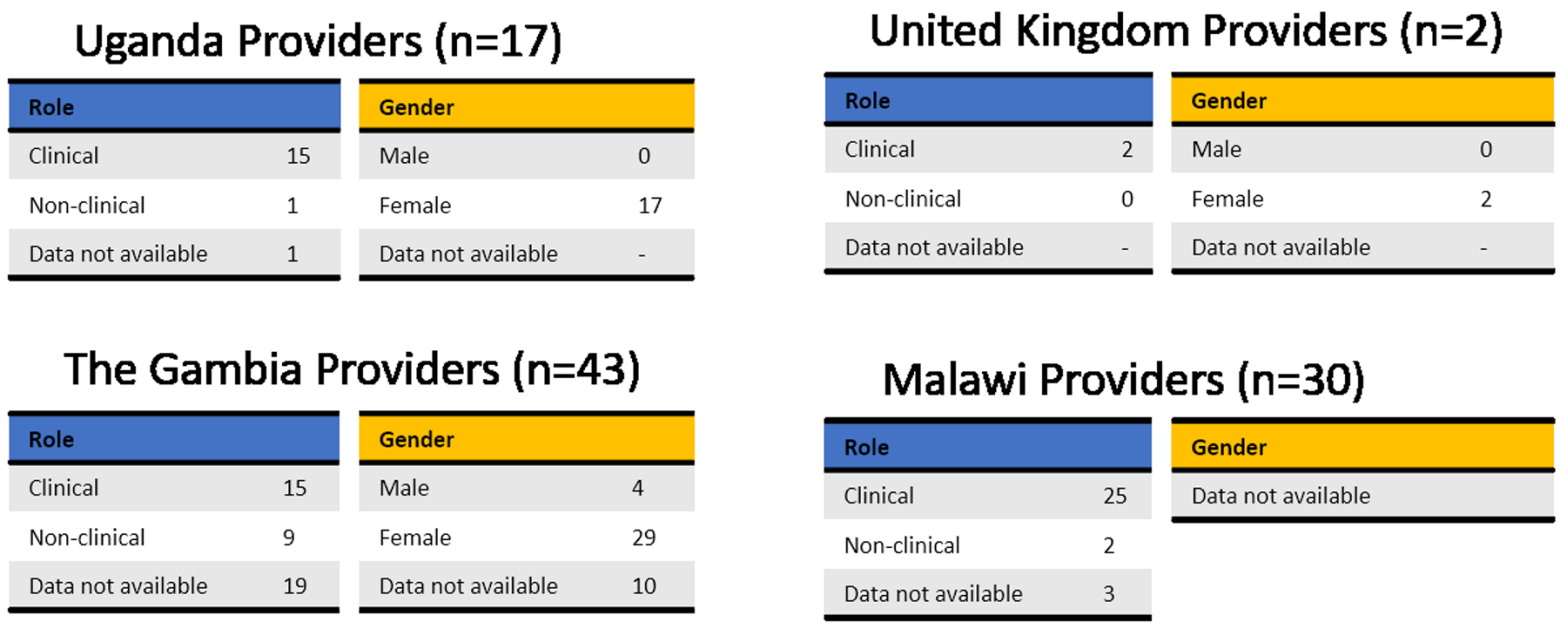
Demographic data of providers grouped by country. Potential providers with a range of roles took part in this study. Tables are based on the available data only, as not all participants completed the questionnaire.

Potential users were selected to cover a range of ages, educations, religious beliefs, family status and experience with contraception ( Figure 4). Male and female potential providers holding clinical and non-clinical roles took part in the study ( Figure 5). 

### Iterative applicator design

Concept research at Design Stage 1 ( Figure 1) led to the creation of a series of prototype delivery systems, which explored different aspects of ergonomics, overall product dimensions, actuation force, feedback on application and potential for self-application. These prototypes, and other visual materials created to support the formative studies, were used to get feedback directly from stakeholders in different countries, as detailed in the section above. The feedback received at each stage helped iterate and refine the design concept to create a final end-user informed prototype. This iterative HCD process is detailed below, and follows the diagram presented in Figure 2 and the timeline presented in Figure 1.

Participants in Uganda (User study 1) were presented with 7 prototype delivery systems and identified positive (ease of use, ease of self-administration, presence of a feedback confirming correct application, small size, easy to understand functionality) and negative (large size, not easy to self-apply, too complicated, scary aesthetic) features of the different prototypes. After handling and discussing the prototypes, participants helped identify four preferred devices out of the seven concepts, with one prototype in particular being preferred by 23 out of 32 potential users and providers because of its intuitive mechanism of action and the ability to be applied it easily with one hand. Overall, the feedback received in User study 1 helped determine the essential features of a successful user experience. The prevailing characteristics of the applicator being as small a size as possible, having a “friendly” aesthetic, and an intuitive actuation mechanism resulted in design refinement and a reduction in the number of prototypes used in subsequent field visits.

Participants in The Gambia and the UK (User studies 2 and 3) were presented with the 4 preferred applicator prototypes (determined in User study 1), to facilitate more focussed and richer feedback. The four prototypes provided participants with a narrowed selection of feedback mechanisms upon application, different small geometries, and a range of actuation methods. Potential users and providers in User studies 2 and 3 preferred the same prototype design as participants in User study 1. The favoured design was perceived as easy and quick to use; one potential provider in The Gambia noted: *“I can do it for somebody, and I can do it for myself”*. In The Gambia, when others observed fellow participants using this particular prototype, appreciative comments, loud cheers and even applause were noted. Specific features of other prototype designs were also recognised as desirable by Gambian and British participants, including smaller size, presence of a feedback mechanism providing visual confirmation of correct administration, and the need for a greater actuation force to provide confidence that an application has been performed correctly. The desirable features identified during User studies 1, 2 and 3 were combined to provide a refined concept for the delivery system. This was subsequently manufactured and presented to potential users in the UK (user study 4). The presence of both visual and audio feedback in this refined applicator design was appreciated by British participants, but they felt that the visual and audio feedback cues could be amplified, giving them more confidence that an application was performed correctly. Participants noted that the force required to actuate the prototype was too low, potentially risking unintended activation, and suggested a greater force for actuation of the device. The transport/disposal cap included in this optimised prototype was considered too easy to remove and therefore not childproof. Feedback from User study 4, together with lessons learned during the previous user studies, directly informed the development of a final optimised prototype device.

In the final optimised iteration, loud audio and clear visual feedback mechanisms were incorporated to indicate effective actuation of the delivery system. The force necessary to deploy the device was doubled, to prevent accidental activation, and the transport/disposal cap was improved to increase childproofing and prevent accidental exposure after disposal. The device dimensions were finalised to accommodate the number of MNs that would be necessary to provide 6 months’ dose of contraception. The internal mechanism, allowing simple actuation and deployment, was engineered and integrated in the design while always considering the end-user preferences for a small, easy to use delivery system; this did not affect the ergonomics of the previously validated prototype and was informed by the data gathered during the user research and feedback received throughout this study. The optimised final prototype delivery system was tested in Malawi (User study 5) where potential users and providers validated the size of the device and commented positively about its aesthetics. Most providers in User study 5 felt the device could be packaged in a comparable way to other medical products. Transparent packaging, to allow users to view the device, or an image of the device on the packaging were suggested to instil trust in potential users. The presence of visual and audio feedback confirming a successful application was considered important by both potential users and providers, particularly given the painless nature of the application.

During the different stages of development, participants were also asked, with the help of visual renderings, if they would prefer the device to have an obvious ‘medical product’ appearance, or an aesthetic totally removed from the clinical environment. Some potential users felt that a colourful device that shared a similar appearance to a make-up accessory would be easy to conceal if taken home, but most potential users and providers expressed a preference for a medical-looking device, with a medical aesthetic deeming to help instil trust in the product. The final prototype reflected this; it was designed with clean lines and it is white apart from the visual feedback indicator. An option to apply a custom label to the top of the applicator was also integrated into the design. This provides a simple way of adapting the appearance of the product to a wide range of users and settings.

### Iterative development of the instruction leaflet

Providing clear and reassuring instructions is key to a product’s success, even though it is an aspect of usability which is often overlooked. Therefore, significant effort was put into developing an instruction leaflet that could be easily understood by all potential users, irrespective of their background. Concept research led to the design of three versions of the instruction leaflet, which were presented to participants in User studies 1, 2 and 3. One version looked more clinical, with precise imagery and detailed text describing the application process. The second version contained simple, cartoon-like illustrations of the application process, with numbered steps as the sole text. The third version was pictorial-only, with detailed illustrations outlining the application steps with colour used to provide emphasis and explain the application process.

Participants felt that the more detailed text instructions were very clear but maybe more suited to health professionals, while the visual-only instructions were perceived to be more suited to women that did not like reading or were illiterate. As one provider in Uganda explained: *“For women, this one [colour coded images] can work. But for us, this one [detailed writing]”*. Generally, participants considered a combination of words and images as the best option, being suitable for all users, no matter their level of education.

A new instruction leaflet was produced in response to this feedback. In this iteration, emphasis was given to the step-by-step visual instructions using a combination of detailed illustrations and use of colour to highlight key elements of the application process. Each illustrated step was also detailed with text, to allow users to correctly apply the product by reading the text and/or looking at the imagery. This improved instruction leaflet was subsequently presented to participants in Malawi (User study 5), who considered it appropriate. However, both providers and users suggested that the instructions need to be available in multiple languages as appropriate to the location (e.g., English and Chichewa for Malawi).

### Anatomical site of application

**Figure 6.**
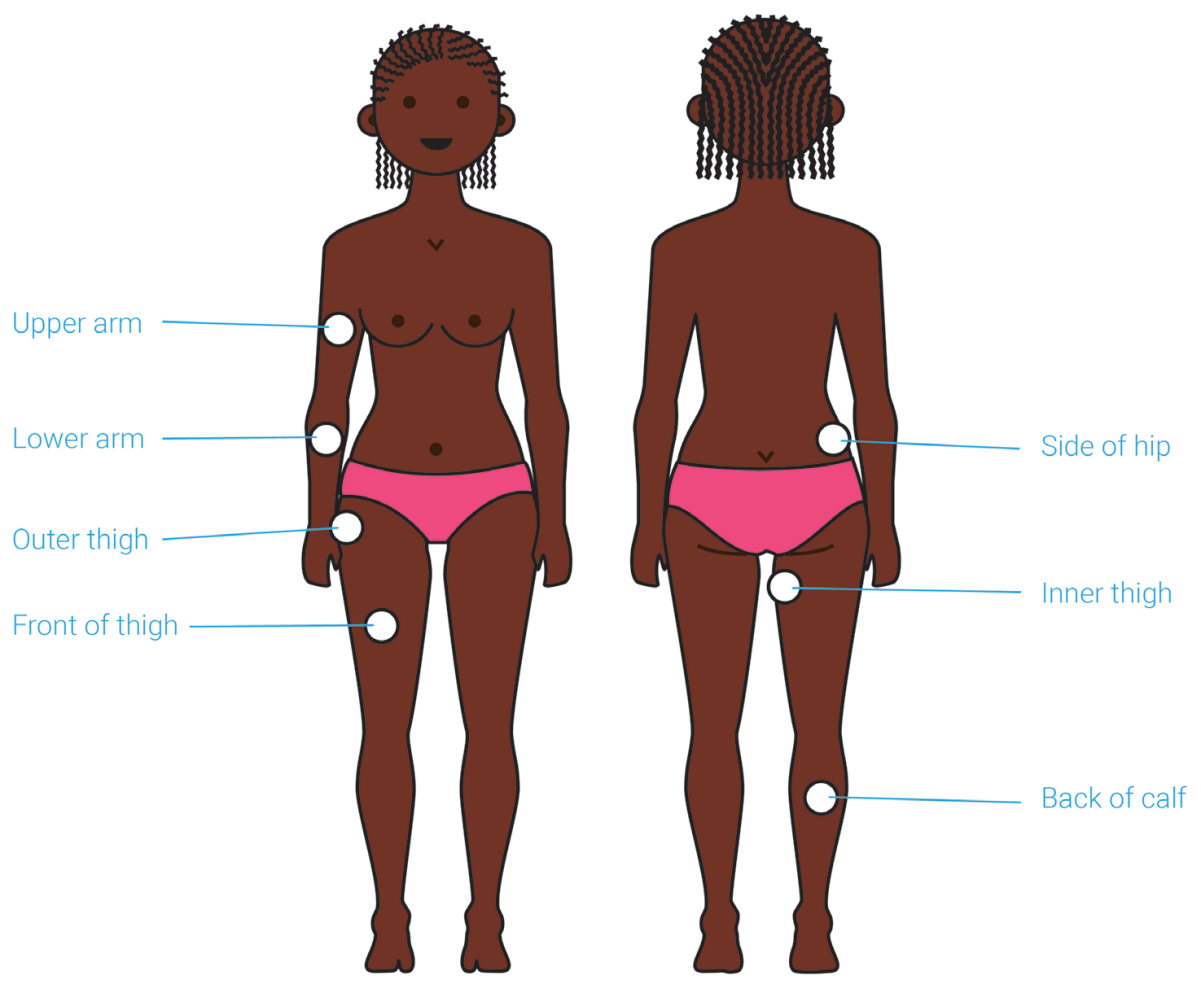
An illustration presented to participants to capture their views on the preferred anatomical site of application. The suitable anatomical sites for application are highlighted on this illustration. Participants were asked to identify their preferred site and explain their choice. This exercise helped identify the upper arm and front thigh as the preferred anatomical site for application of the proposed contraceptive delivery system.

Participants feedback was sought on the proposed anatomical sites of device application, with the help of visual renderings ( Figure 6). The concept was developed for application to ‘fleshy’ areas of the body, such as the thigh or the side of the hip, and so a limited number of options were offered.

The thigh (if self-applied) and the upper arm (if applied by a provider) were identified as the preferred anatomical sites for administration by most participants at all stages of product development. As one provider in Uganda explained: *“On the thigh if it [is] self-administered. But if it can be administered by a nurse, it can be the arm.”* These sites were perceived as easy to access, easy to conceal with clothing after administration (Potential user, The Gambia: *“If people don’t see it, then it’s where I’m going for”*), less painful *(*Potential user, UK: *“I don’t think I would like it on the side of the hip coz I think that would hurt more”)*, and familiar due to the fact that they have experience with other medicines that are applied to these body sites.

### Scenario for contraceptive provision

**Figure 7.**
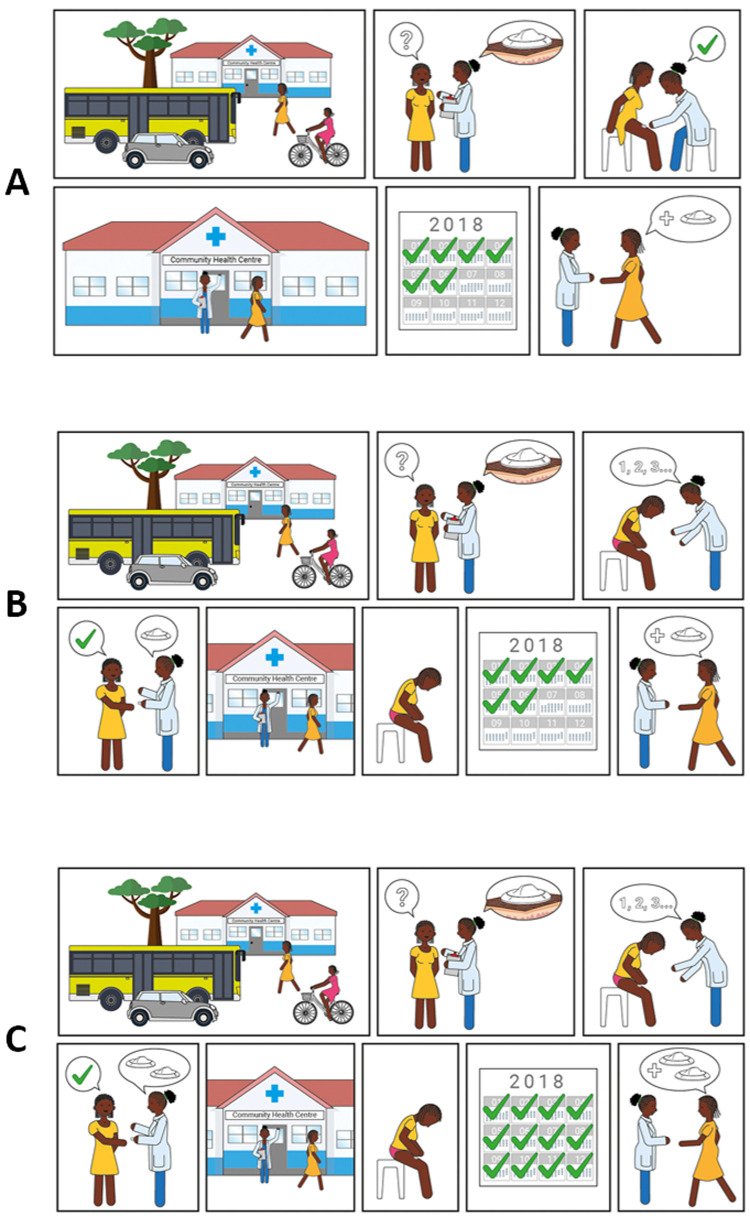
Illustrations used to present different access scenarios to participants. Scenario **A**) Access is always via a health centre, where a health care professional applies the contraceptive. Scenario **B**) Access is via a health centre, where a healthcare professional trains the user on how to apply the contraceptive correctly; self-applications are then performed when desired. Users must return to the health centre to receive subsequent contraceptives when needed. Scenario **C**) Access is via a health centre, where a healthcare professional trains the user on how to apply the contraceptive correctly and gives them 2 devices. Users do not need to return to the health centre for a second application when the contraceptive effect of the first application lapses.

Three different scenarios to access the contraceptive solution were presented to participants ( Figure 7). The majority of potential users in The Gambia and Malawi felt the device should be distributed by hospitals and clinics and administered by a health professional (scenario A) to ensure appropriate application of the device and privacy (Potential user, The Gambia: *“Example, if you have a husband, he travels outside The Gambia and you are taking this device, what will your neighbours say? The husband is not here and she’s taking contraceptive”*). Potential users in Uganda expressed an interest in obtaining the device through hospitals and clinics prior to self-administration at home, but this was caveated with the requirement for practical demonstration by a healthcare provider upon first use (scenario B). Potential users in the UK preferred access to a stock of the new product via health clinics or pharmacies for subsequent self-application at home (scenario C) (Potential user, UK: *“It’s [a] hassle making appointments and stuff if you could just have a stock that you have at home that you apply every 6 months”*, Potential user, UK: *“I’d want to be shown how to do it first and then if it doesn’t actually seem that difficult to do then I’d be happy to do it on my own then after that… Even if they had like a dummy one so without the actual ingredient in it to show you how to do it because it is a new technique and then give me the thing”*).

Potential providers and organisations in Uganda and The Gambia expressed a preference for administration of the contraceptive by health professionals (scenario A). There were multiple reasons for this including correct application (Provider, The Gambia: *“If they take it, some women, they cannot apply properly”*), appropriate storage (Provider, Uganda: *“Some mothers have homes which cannot keep that safely*”) and disposal of the device, maintaining regular healthcare appointments (for review and accurate completion of medical records), and concerns over women distributing unused devices to others. Potential providers in Malawi felt the potential to self-administer was very attractive for women and most felt that women would be capable of self-administration, albeit with training or experience (scenario B); only 2 out of 30 providers said women would not be able to self-administer. There was however some disagreement about whether women would want to self-administer, a view that would be dependent on the woman and/or her partner. Several advantages to self-administration were noted by providers in Malawi, in particular the need for fewer trips to hospital and the resulting decrease in workload for the clinics. However, as in Uganda and The Gambia, some concerns were raised about incomplete medical records, incorrect and unsafe administration of the contraceptive, inappropriate storage and disposal, and the opportunity for misuse and abuse.

### Duration of action

**Figure 8.**
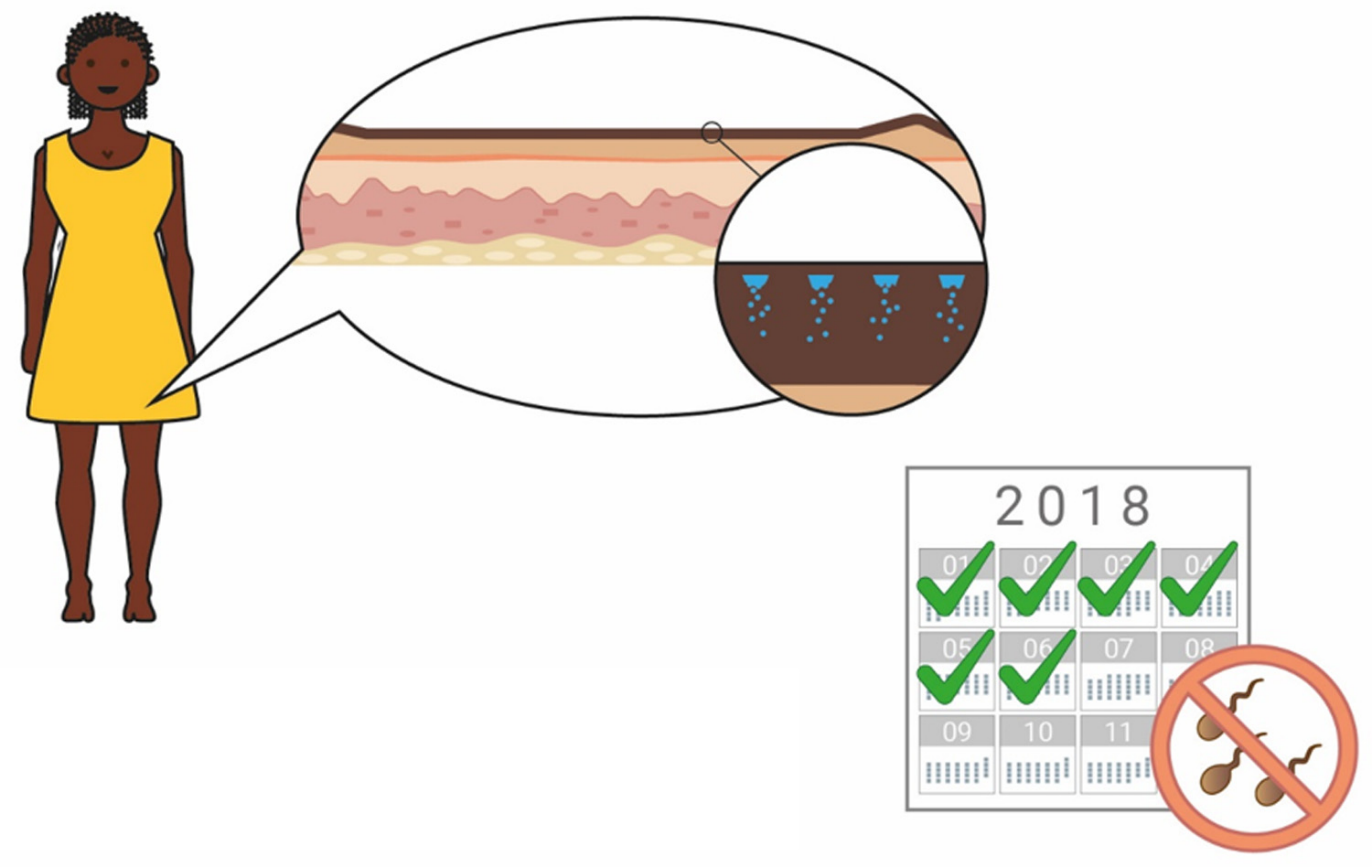
An illustration used to explain the concept to participants. The concept of a 6-month duration, reversible contraceptive based on biodegradable microneedles was explained to participants with the help of this illustration.

When the concept of a long-acting contraceptive delivered by biodegradable MNs was explained to participants, a 6-month duration of action was used as an exemplar ( Figure 7 and Figure 8). When asked to comment about this duration, potential users, providers, and organisations generally found this duration attractive, predominantly because it would prevent repeated visits to the clinics (Provider, The Gambia: *“Coming to the health facility every month will be a burden, but if they have something like six months and upwards, it will be better”*). It was also noted by many stakeholders that a method of contraception with a 6-month duration is not currently available on the market, and this would provide potential users with more choice (Potential users, UK: *“It’s quite nice because it’s”…”in-between other things that are already available”*). Some participants in Uganda, The Gambia, and the UK highlighted the value of a shorter acting ‘trial’ option to determine efficacy, acceptability, and tolerability before committing to a longer duration of action, particularly as this method of contraception, unlike an implant, cannot be reversed once administered (Potential user, Uganda: *“If you would say, ‘First have a smaller interval’ then they see how they can go*”, Potential user, UK: *“I’d wanna trial the trial period before I committed to 6 months of having something because like side effects and stuff is such a big thing with contraception”*). However, it was also noted that having different versions of the product with different durations may cause confusion. Potential users in Malawi commented that, most importantly, the duration that is specified on the user instructions must be guaranteed.

The inability to terminate (i.e. remove) the contraceptive effect once administered caused some concerns with potential providers in Malawi, as they felt potential users may not understand this. Many providers considered this to be acceptable if carefully explained to potential users, while some considered this to be problematic. The majority of potential users in Malawi did not identify this as an issue.

### Return to fertility

The return to fertility following the use of the proposed MN-based hormonal contraceptive delivery system explored in this study would not be immediate after the 6-month protective period. We explored participants’ views on this matter and asked them what interval of time would be acceptable to return to fertility.

 Participants identified the time to return to fertility as very important (Provider, The Gambia: *“Even if you are not using any contraceptives and even if physically normal state… it may, may take you months before they get pregnant. However, if a woman takes contraceptive, the woman is two, three months they are not pregnant, they think it’s still the contraception”).* However, there was no consensus amongst participants on the most acceptable time for a return to fertility, with answers ranging from immediately to up to one year. It was noted that the answer might depend on the individual user and their circumstances, but a large proportion of women would prefer a relatively rapid return to fertility, in the range of 0–3 months.

### Identified benefits and concerns

The concept of a new MN-based progestin-only hormonal contraceptive was generally viewed positively by all participants. Some of the advantages identified by participants included the pain-less, blood-less, user-friendly and time-saving nature of the application (User, Malawi: *“This device is not frightening because the needles are small and hard to see unlike the other injections whereby the syringe is big and it’s frightening when you see it“*; Provider, The Gambia: *“I believe that they would prefer to take this one… it’s easy, it’s friendly, it’s user-friendly. … And at the same time, they’re having no pain. Most of these women, they are scared of this pain”*; Provider, Malawi: *“It is user friendly. Even a school drop-out can manage to use it”*), the intermediate duration of action, the potential for greater compliance compared to other methods, the potential for self-administration, the discreet nature of this solution (Provider, Uganda: “ *Some husbands don’t consent to family planning. So if this wasn’t able to be seen, then it’s okay”*), the reduced risk of needle-stick injury and cross infection (Provider, The Gambia: *“As a nurse, one of the other benefits is you’ve got a reduced risk of sharps injury”*), and the biodegradable nature of the system, i.e. no need for surgical removal.

Overall, the proposed method was recognised as a means to offer women more choice and was viewed positively and many potential users expressed an interest in trialling the new method when it becomes available. (Users, The Gambia: *“So many will go in for it!”, ”If this one is available the other methods will not be used!”, ”When will you get this? I want it.*”). Potential users that were unhappy with their current method of contraception were particularly enthusiastic. Potential providers noted that painless application and the potential for self-administration are particularly attractive features that could be empowering for women and they would recommend such a method to potential users, although more detailed information would be required when the product becomes available (Provider, Malawi: “ *People will opt for it because it is pain-free as well as the duration… even if it is me, I can opt for it”*).

The majority of the concerns that were raised by participants related to possible side effects (especially bleeding abnormalities) that are common with all hormonal contraceptives (User, The Gambia: “ *Does it not have side effects? Cos I’d like that, no side effects.”*). The novelty of this approach was also identified as a potential source of apprehension, with thorough testing being recognised as essential to engender trust in the product and confidence in its use. Providers said that word of mouth is a common factor in making contraceptive choices in SSA and it would take time to build this trust with potential users (Provider, Malawi: *“This new development will be hard to intercept for the first time but with time those who will take it will help in disseminating the information to other people in their communities, especially in their various groups….slowly people will be familiar with it”*) and women commented on the importance of experience in engendering trust (User, Malawi: *“Once six months elapses without conception, I will know that it is good”*). There was also some concern over potentially being the first users of the new product (Provider, Malawi: *“You’ve said that the method has not yet been implemented anywhere in the world and you are doing a research which means if it is to be implemented, it will also start here in Africa - so people would be afraid”*).

Generally, providers believed that the concerns of potential users may be addressed through education and awareness, and suggested training packs and visuals would be important to help explain the new concept to women. The irreversible nature of the contraceptive effect for the duration of action was identified as potential issue, and providers felt that this feature would need to be clearly communicated to users prior to administration. Potential users were more concerned about the fate of the dissolved material in the body, the potential detectability by others (User, Malawi: *“It should not leave a mark because even now my child keeps on asking me questions about the mark I have on my arm”*) and the cost of this new method.

In Malawi only, even though lack of pain was recognised as a benefit in most cases, some providers and users were concerned about the painless nature of the method, noting that women may want to feel pain to provide reassurance that an application was successful and that it would be effective (Provider, Malawi: *“Here in Malawi most people believe that the medicine is very effective and powerful when they feel pain and bleed after they are injected….so if it is painless they will think that the medicine is not very effective”*, Users, Malawi: “ *We should feel pain a bit as an assurance that the medicine has entered the body”*, *”It could be that this method won’t have such effect as other injections that make us take painkillers after being injected”*).

The name of the product was also identified as a potential barrier as the term ‘needles’ is associated with a metal product and this may provoke negative connotations with respect to deposition in the body (Providers, Uganda: “ *Of course, if you mention the needle that is going to remain in your body”*, “ *They get scared”*) *.* Some participants also raised a concern related to the storage and transport conditions of the product, more specifically the inability of a potential user to effectively store a product at home if it requires refrigeration and concern that a device could be inadvertently deployed in transit or storage.

Several additional questions were raised by providers, including the time taken to establish contraceptive effect following administration, the method of disposal, and suitability of the product for under 18s, breastfeeding mothers or women being treated for HIV infections.

## Discussion

In this study, the HCD approach used to develop a new MN-based progestin-only hormonal contraceptive delivery system targeted at women who have an unmet need for contraception is described. We anticipate that the involvement of the intended end users and other stakeholders during all phases of product development has facilitated the design of a product that is more likely to be accepted and adopted in LMICs.

This study is consistent with other comparable studies ^12, 19^, indicating that new methods of contraception are welcomed in LMICs to increase the range of available options. A method that requires minimal training would be more accessible and with this in mind our research team have developed a user-informed device that is simple to use and can potentially be self-administered.

Throughout all stages of product development, participants feedback was used to identify the most desirable features of the MN delivery system, and to iteratively improve these characteristics according to users’ preferences. Initially, participants were shown many prototypes with different sizes, modes of action and feedback mechanisms. The preferred features at this stage were: small size, ease of use, unalarming appearance, and clear feedback on application. A refined concept prototype was developed according to users’ preferences, and participants identified features of this prototype that needed improvement. In particular, participants viewed the presence of a visual and audio feedback mechanism as a very positive feature but suggested that the visual cue and sound could be amplified to increase confidence that the application was performed correctly. Participants also expressed a preference for the force required to actuate the device to be increased, to avoid unintentional activation, and for the transport/disposal cap to be made more difficult to remove and therefore more childproof. This feedback was integrated into the final prototype, which was validated in a final round of user testing. 

Participants’ views were also sought on additional aspects of the suggested MN-based progestin-only hormonal contraceptive, such as anatomical site of application, mode of access, contraceptive duration, and time to return to fertility. The results emphasised that the preferred site of application for such a device could depend on who was performing the application: women would prefer to self-administer the MN-based contraceptive to their own thighs but would prefer the upper arm as a site of administration if the device was applied by a health care professional, as they wouldn’t be comfortable showing their legs, in particular to male health care providers. In both cases, the ability to cover the treated site with clothing immediately after application was deemed crucial by participants, since discretion can be a key factor in choosing a contraceptive method in LMICs ^20, 41^. This was considered when refining the product design, ensuring that this delivery system is suited to application at either of these two body sites (including being deployed by one hand) and is as discreet as possible.

Regarding access to this new method, some women in LMICs highlighted how going to the clinic for contraception is a good opportunity to discuss their sexual health with a professional, while others would prefer to be given the device to apply in the comfort of their home, in their own time. Some women thought that storing/using the contraceptive at home could be an issue if they wanted to conceal it from partners/family members, while for women whose partners are involved in contraceptive decision making this would not be an issue. Whilst recognising the advantages of self-administration, many of the health care professionals in the LMICs preferred the application to happen in a clinical setting, to maintain accurate records and check on the women’s health during the appointment. Both health care providers and organisational stakeholders in LMICs were also worried about women sharing or selling the contraceptive if they were provided one to take home. Women and providers in the UK were less concerned about access to the device, suggesting it should be available in health clinics and pharmacies for home application. The proposed delivery system has the flexibility to be applied by a health care professional or self-administered and could be accessed in different ways in different countries, to reflect different socio-cultural norms. The final optimised device also has the flexibility to have an unassuming, medical look or a more colourful, unrevealing appearance.

Interestingly, users’ opinions on duration of action and return to fertility were mixed and mostly dictated by the women’s personal experiences and needs. Women with no or fewer children found the proposed 6 months duration attractive as a mean to delay their first pregnancy or space their children according to their preferences; a return to fertility in the range of 0–3 months was considered acceptable in these circumstances. Women with many children expressed an interest in even longer durations, as a mean to stop having children; return to fertility was not a big concern for these women. Users who previously experienced problems with long-acting methods mostly preferred a shorter duration with a quick return to fertility. While the proposed duration for this new MN-based contraceptive is 6 months, the manufacturing method provides flexibility, allowing for a range of durations of action. In each case the return to fertility would need to be assessed during pre-clinical and clinical testing.

Users identified many possible advantages of the new proposed MN-based contraceptive delivery system, including benefits related to the minimally invasive nature of MNs (pain-free, blood-free, discreet), the intuitive operation of the applicator (quick, easy, user-friendly, requiring minimal or no training), and the proposed 6 months duration (fewer visit to the hospital, fills a duration gap in the market between injectables and implants).

Users expressed a range of concerns related predominantly to side effects. This is consistent with other studies on new proposed contraceptive devices ^20, 42^. Participants frequently inquired about the side effects of the device, and repeatedly mentioned bleeding changes, weight changes, and the fear of permanent infertility as particular worries regarding any contraceptive product. Whilst the proposed device has not yet entered the clinical stage of development and we are not in a position to confirm side effects, given the requirement for ovulation inhibiting blood levels of contraceptive we anticipate a comparable hormonal side effect profile to LNG subcutaneous implants ^43^. Another concern that was repeatedly raised was the name of the new technology. Users did not like the word ‘needles’ due to an association with pain. Careful naming and branding of the technology, alongside education, information, and discussions with healthcare professionals, will be critical to create trust in the new product.

In conclusion, the concept of a new MN-based hormonal contraceptive delivery system was generally viewed positively in the visited LMICs, but some potential user concerns were identified. This product was iteratively developed using a HCD approach, meaning that potential stakeholders identified features of their ideal product and influenced the final design of the delivery system. This prototype is now being evaluated in pre-clinical testing. Having followed this approach, validation of the final product through a summative study, which will in time be required for the certification of the MN contraceptive delivery system, should be rendered a more straightforward exercise. The team is integrating the valuable feedback received by participants in this study in the final design of this novel minimally invasive hormonal contraceptive delivery system, that will be taken forward for pre-clinical testing.

## Data availability

### Underlying data

Full qualitative transcripts are not available for ethical reasons because even after removing directly identifiable information such as names and addresses, participant identity may be difficult to fully conceal, and research locations may remain potentially identifiable, presenting a risk of deductive disclosure. However, codebooks and relevant excerpts of transcripts are available from the authors on reasonable request. Requests should be sent to the corresponding author at HughesML@cardiff.ac.uk or to BirchallJC@cardiff.ac.uk. Requests will be granted to researchers for the purposes of comparative analysis, upon approval from relevant ethics committees.

### Extended data

Figshare: Supplemental material manuscript for "Human-centred design of a new microneedle-based hormonal contraceptive delivery system". https://doi.org/10.6084/m9.figshare.14697393 ^36^.

This project contains the following extended data:

- Supplemental material manuscript 13233.pdf (participant information sheets, consent forms, questionnaires, and FGD/SSI topic guides used)

Data are available under the terms of the Creative Commons Attribution 4.0 International license (CC-BY 4.0).
