# Supplementary material for: Human-centred design of a new microneedle-based hormonal contraceptive delivery system
Source: Gates Open Res. 2021 Dec 1;5:96. Originally published 2021 Jun 17. [Version 3] doi: 10.12688/gatesopenres.13233.3 (PMC9020198; doi:10.12688/gatesopenres.13233.3)
Supplement: Peer review history

# Peer review: Reviewer response for version 3

**DOI:** 10.21956/gatesopenres.14731.r31912

**Published:** 2022-04-19

**Authors:** Aluisio J.D. Barros

**Affiliations:** 1 International Center for Equity in Health, Universidade Federal de Pelotas, Pelotas, Brazil

**General comments:**

 The manuscript presents the process of perfecting the design and characteristics of a new microneedle contraceptive device. A human centered approach was used to design a multicountry qualitative study. The main objectives were to direct the design of the new device, the duration of its effect and guide the contents and layout of the user guide.

 The methods used, even considering the qualitative design, were very flexible with many aspects defined by convenience, such as the type of interview (focus group or semi-structured interview), source of participants, numbers, selection of partners.

 The results are described in very general terms, in a narrative fashion, with nearly no organization or tabulation of the aspects studied and respective preferences. Notably, no clue about what the device in study looks like is given to the reader. As a consequence, the reported preferences by participants sound a bit obvious like a choice of a smaller device that is easy to use or a user guide that is an illustrated step by step document.

**Specific comments:**

1. Abstract – To say that more than half of women with an unmet need for contraception live in LMIC is not informative. Nearly 85% of the world population live in LMIC. The same statement is repeated in the introduction.
2. The frequent use of acronyms makes the text difficult to follow. The authors go on accumulating abbreviations like MN, HCD, FGD, SSI. I suggest that only easily recognized acronyms are used (e.g. LMIC).
3. There is a detailed presentation of methods, but we are left with the impression that several important aspects such as sample size, selection of participants, type of interview were essentially based on convenience. I wonder what impact this may have had in the results.
4. The section “Iterative applicator design” is rather awkward to read, as the text goes on describing theoretical advantages sought – like obviously small size and ease of use – but without the reader having a clue of what the device is, looks like or does. If the device cannot be presented due to commercial issues, I think it would be easier to just present a table with the main characteristics found relevant by the participants and how each version scored.
5. By the end of the section, audio and visual feedback mechanisms are mentioned and we are kept wondering what the size of this device is, or what it looks like. Not even an approximate size and format is presented in the text.
6. Having said that, I was curious to know about the likely situation of people not finding this device usable at all. How many of the women were not convinced by the proposed device?
7. Again, the development of the instructions is based on a presentation of preferences on general characteristics – text or illustration based – without presenting specific details. It is not surprising that users would prefer a clearly illustrated step by step guide.
8. I found the general preference of users for the device to be applied by a health worker in opposition to the user friendliness and ease of use stressed in the benefits and concerns section. Again, having no idea of what the device looks like or how it is used does not help.
9. Later in the section statements like “So many will go in for it!”, ”If this one is available the other methods will not be used!” make me concerned about the lack of a comparison alternative method. People are very keen to agree and please the interviewers in focus groups. I myself had this kind of experience where participants were very positive about a device presented to them, and later, after getting one, they never used it.

Is the work clearly and accurately presented and does it cite the current literature?

Yes

If applicable, is the statistical analysis and its interpretation appropriate?

Not applicable

Are all the source data underlying the results available to ensure full reproducibility?

No

Is the study design appropriate and is the work technically sound?

Yes

Are the conclusions drawn adequately supported by the results?

Partly

Are sufficient details of methods and analysis provided to allow replication by others?

No

Reviewer Expertise:

Epidemiology, global health, RMNCH

I confirm that I have read this submission and believe that I have an appropriate level of expertise to confirm that it is of an acceptable scientific standard, however I have significant reservations, as outlined above.

# Peer review: Reviewer response for version 3

**DOI:** 10.21956/gatesopenres.14731.r31499

**Published:** 2022-01-14

**Authors:** Rebecca L. Callahan

**Affiliations:** 1 FHI 360, Durham, NC, USA

I update my review status to “Approved”. However, I do encourage the authors to review the abstract and perhaps revise to include the key results of the design activities. As it currently reads, the abstract results describe more of the methodology rather than what emerged from the HCD process.

Is the work clearly and accurately presented and does it cite the current literature?

Partly

If applicable, is the statistical analysis and its interpretation appropriate?

Not applicable

Are all the source data underlying the results available to ensure full reproducibility?

No source data required

Is the study design appropriate and is the work technically sound?

Yes

Are the conclusions drawn adequately supported by the results?

Yes

Are sufficient details of methods and analysis provided to allow replication by others?

Partly

Reviewer Expertise:

NA

I confirm that I have read this submission and believe that I have an appropriate level of expertise to confirm that it is of an acceptable scientific standard.

# Peer review: Reviewer response for version 2

**DOI:** 10.21956/gatesopenres.14599.r30973

**Published:** 2021-08-23

**Authors:** Rebecca L. Callahan

**Affiliations:** 1 FHI 360, Durham, NC, USA

This article describes a novel approach for assessing and incorporating user preferences into the design of a new contraceptive delivery system.  The authors’ adaptation of human centered design for contraceptive R&D is innovative and provides a useful model for others working in new contraceptive product development. The inclusion of multiple study sites and types of participants – potential users, providers, and other stakeholders – strengthens the findings. While the authors cite other studies that have attempted to evaluate user preferences for new contraceptive technologies, they might consider reviewing the following paper, which also evaluated potential interest in and preferences for a microneedle (MN) patch for contraception: Callahan *et al.* (2021 ^1^).

 While the paper presents an overview of the human centered design process used to iteratively devise the preferred MN contraceptive, it would help to describe in more detail some of the steps and the actual concepts developed during the process. Under “Concept development” the authors state that, “the concept of a new MN-based progestin-only hormonal contraceptive delivered by an applicator was developed by the team.” Can you provide a description of this concept? Maybe add an image? Also, who is “the team”? The authors? Adding images of the product iterations would help the reader better understand what study participants were reacting to and which product attributes changed over the course of the research.

- In the second paragraph of the Methods section the acronyms "FGDs" and "SSIs" should be defined (they are defined subsequently, but not at first use). The authors might also consider adding a table describing the number of FGDs and SSIs conducted in each country. 
- The Study Design section could be strengthened. It would help to clarify the original data collection plan and what took place. Was it determined at the time of data collection which method would be used? What is meant by “pragmatic qualitative approach” and “dynamic nature of discussion”? 
- Was ethical approval obtained in Uganda? It is not listed in the manuscript.
- Why not show images of the prototypes produced by Maddison Ltd? Also, were these prototypes based on ongoing MN product development research? If so, it would help to describe this. 
- Under Sampling and Recruitment, who are “local gatekeepers”? How were they identified?
- The study generated many results. To more clearly relay what concepts/prototypes participants were responding to in each phase of data collection and to better understand their reactions, it would help to show an image of the prototype and list the specific attributes of the product. Perhaps a figure illustrating the changes in the prototype over the course of the study resulting in the final product design. Images would help.
- What does “friendly” aesthetic mean?
- The term “applicator prototypes” is a little confusing since some MN products include an external applicator. Can you clarify what you mean?
- Though it becomes clear with further reading, it would help to define “actuation methods” up front. How is this different from “deployment”?
- In the second paragraph on page 9, you state that the device dimensions were finalized to accommodate the number of MNs that would be necessary to provide 6 months’ dose of contraception? How are these dimension known? This relates to my prior question about whether this study is associated with ongoing R&D efforts and, if so, how. 
- Why not show an image of the “final optimized iteration”?
- What does “medical-looking” mean? Images would help.
- As described above, it would help to describe more the baseline requirements/underlying assumptions of the 6-month MNP (perhaps based on ongoing R&D work), show the initial prototype, and then show the iterations that were used in the subsequent studies.
- Can you explain what does it mean to “validate” the prototype?
- Some results seem to be presented in the discussion that do not appear in the results section.

Is the work clearly and accurately presented and does it cite the current literature?

Partly

If applicable, is the statistical analysis and its interpretation appropriate?

Not applicable

Are all the source data underlying the results available to ensure full reproducibility?

No source data required

Is the study design appropriate and is the work technically sound?

Yes

Are the conclusions drawn adequately supported by the results?

Yes

Are sufficient details of methods and analysis provided to allow replication by others?

Partly

Reviewer Expertise:

contraceptive research and development; global family planning; user preferences

I confirm that I have read this submission and believe that I have an appropriate level of expertise to confirm that it is of an acceptable scientific standard, however I have significant reservations, as outlined above.

## References

[1] : Optimizing the design of a contraceptive microarray patch: a discrete choice experiment on women's preferences in India and Nigeria. *Reprod Health* .2021;18(1) : 10.1186/s12978-021-01113-y 67 10.1186/s12978-021-01113-y 33752700PMC7986031

# Response

**Published:** 2021-11-25

**Authors:** Benedetta Gualeni

**Affiliations:** Cardiff University, UK

We thank the reviewer for their positive comments relating to the important contribution that this article will make. We address specific comments in turn below.

 Regarding the initial comment, the reviewer shared a recent paper that might be cited in the article. This is a very interesting article and we thank the reviewer for sharing it. The reviewer might not be aware that our manuscript was submitted to Gates Open Research on March 15 ^th^ 2021, before the referenced article was published (available online from March 22 ^nd^ 2021), and so we did not have knowledge of this body of work at the time of submission. Due to our submission date being before the publication of this article we presume that it is not appropriate to include reference to it, at this later stage.

 Nevertheless, our work is in line with what is suggested in the referenced article, i.e. in developing our contraceptive MN-based product we are incorporating the perspective of potential end users to maximise its chance of being adopted in the future. Also, some of our findings via FGDs and SSIs are in line with the results from DCE described in the referenced paper. In particular, potential end users also highlighted to us the preference for minimal or no effect on their menstrual cycle while using the contraceptive; they were particularly interested in the contraceptive effect lasting 6 months and in a patch size as small as possible.

 The effect of our product on the menstrual cycle will be evaluated in future clinical studies and we acknowledge that any possible effect on menstruation might deter some potential users to adopt this new solution. Nevertheless, adding a new method that has the potential to be self-administered might cater for the needs of some potential users whose needs are not currently met.

 In response to the following comment, and also to comment 1 from the other reviewer we have added the following paragraph to the manuscript:

 “The initial concept was to develop a long-acting, reversible, MN-based, progestin-only hormonal contraceptive, that is delivered by an applicator, can be self-administered (or administered with minimal training), is stable at high temperatures and high humidity, and can be mass produced at low cost. An assortment of potential products, with different features (Design Stage 1, Figure 1) were therefore developed. These initial product designs were informed by the published literature, the opinions of stakeholders/‘experts’ with experience of developing and/or using FP in LMICs and the multidisciplinary research team, which includes product designers, engineers, pharmaceutical scientists and polymer scientists.”

 As we have also mentioned in reply to point 13 from the other reviewer, we are unable to provide any further details at this stage due to a potential IP disclosure limiting our ability to further develop the product. Once IP is more secure a technical paper describing the development of the actual MN product will follow. We have added the following paragraph to the manuscript to clarify this issue:

 “At this stage we are not able to share any images of the prototypes, as publishing them will constitute a prior art disclosure that will inhibit our ability to protect the novel mechanism used within the applicator.”

 In response to the first bullet-point, thanks for spotting that FDGs and SSIs were not defined at first use. This has now been corrected in the manuscript. We have considered whether a table may be useful but do not think it necessary.

 In response to the second bullet-point, the original data collection plan was to gather feedback from as many and as wide a range of potential users, partners, providers, and stakeholders as possible during the data collection period at each stage. FGDs were designed to be our method of choice for data collection as they allowed for more interaction and discussion but, at the time of recruiting, if participants showed a preference for SSIs we accommodated them. Also, if we would miss out on data waiting for sufficient numbers for a FGD to take place then we offered the opportunity for a SSI instead thereby capturing data that might otherwise be lost.

 We ran this study in line with the original data collection plan but in a pragmatic manner (pragmatic qualitative approach) - in other words while trying where possible to stick to the original plan we were conscious that practical issues may impact on recruitment and data collection and so we were prepared to be flexible where needed - for example by offering SSIs instead of FGDs. We took the approach that rather than turn down data collection opportunities because, e.g., we had reached our desired numbers or had lots of people from a similar background, we would make the most of all opportunities given the limits of our logistics.

 By “the dynamic nature of FGDs” we mean that discussion is more open and less directed than during an SSI, going beyond the topic guide. This interaction between participants really allows data collection to be more participant-driven than researcher-driven, with participants explaining, questioning and justifying their viewpoints to each other, and this enables a deeper understanding of the participants’ views and perspectives, often resulting in unexpected findings being revealed.

 In response to the third bullet-point, the ethical approval obtained for Uganda is the one listed as “Mountains of the Moon University Research Ethics Panel (MMU/DPGSR/061218)”. To clarify this we added “Uganda” in brackets before the reference number.

 In response to the fourth bullet-point, as already stated above, we are not able to share any images of the prototypes at the moment, as publishing them will constitute prior art that will interfere with securing IP for the novel mechanism used with the applicator. The applicators are currently being developed by our research team, and are innovative in terms of shape, manufacturing, deployment, mechanism of action and duration of action. They are absolutely based on our product development research.

 In response to the fifth bullet-point, the gatekeepers were identified through our links with charitable organisations and academic collaborators and they are either listed as authors (when they also contributed to data collection) or acknowledged in the acknowledgment section (when their role was solely that of gatekeepers).

 In response to the sixth bullet-point, even though we agree that showing different prototypes and how they were modified in response to participants and ultimately consolidated in a final design would be beneficial, unfortunately we cannot show any pictures due to IP reasons as stated above. 

 In response to the seventh bullet-point, participants perceived larger prototypes as “scary”. Other published studies have also suggested that sharp corners might contribute to create wariness in a product. Also, certain colours might be perceived as frightening. A “friendly” aesthetic in our final design means a design with a small shape, rounded corners, and neutral colours to help reduce lack of trust into the device due to its physical appearance.

 In response to the eighth bullet-point, participants in this study were not shown the final product, composed of MNs and an applicator to administer them to the skin. Only the proposed external applicator prototypes were shown to the participants. These consist of the fully functional applicators without any MNs (just a flat plastic surface where the MNs would be). This allowed participants to handle the applicator prototypes and inform on their preferred characteristics.

 To clarify this issue we have added the following paragraph to the manuscript:

 “The dummy prototypes used in the studies consisted the fully functional applicators without the presence of MNs. This allowed participants to experience the mechanism of deployment, usability, duration of application and feedback from the applicator.”

 In response to the ninth bullet-point, actuation refers to the mechanism by which the applicator is initially activated by the user, while deployment refers to completion of the application process.

 In response to the tenth bullet-point, the present manuscript describes our effort to include users view in the development of a MN product in terms of usability, especially with the aim of self-administration in mind. In parallel to the user studies the team is undertaking technical development of the MN product and we have calculated and manufactured the dimensions of MNs that will facilitate a 6 month dose of contraceptive. The studies described in this paper are both based upon, and informing, the R&D development.

 In response to the eleventh bullet-point, we are unable to provide an image due to the aforementioned IP disclosure issues.

 In response to the twelfth bullet-point, the “medical-looking” aesthetic we presented to the participants meant that the prototypes had neutral colours, without any patterns or colourful images on the casing. Participants themselves used this terminology to mean a more neutral design with colours more typical of a clinical setting or other clinical devices. We have added a clarification of the meaning of “medical-looking” in the manuscript: “(i.e. clean, neutral colours)”

 In response to the thirteenth bullet-point, and also to comments from the other reviewer, we have added this sentence to the manuscript to clarify this point:

 “Injectable contraceptive products provide 12 weeks (3 months) of contraception and implants provide >12 months of contraception to the user. The proposal was therefore to develop a product with an alternative duration of action that would provide users with another contraceptive choice. Based on current technology, a 6-month duration of action is at the technical limit of what can be achieved using a relatively small microneedle array patch, considering drug loading limitations and the preferred size of patch indicated by potential users”.

 In response to the fourteenth bullet-point, to “validate” the prototype means to show the improved version of the prototype to users and confirm that the characteristics that received positive feedback in previous iterations are still liked by participants, while the features changed to reflect users’ preference receive positive comments.

 In response to the fifteenth bullet-point, we reviewed the manuscript and could not find any specific examples of results presented in the discussion that did not appear in the result section. We remain available for further clarifications if the reviewer wishes to provide some specific examples.

# Peer review: Reviewer response for version 2

**DOI:** 10.21956/gatesopenres.14599.r31060

**Published:** 2021-08-11

**Authors:** Maggie Kilbourne-Brook

**Affiliations:** 1 PATH, Seattle, WA, USA

This article details the process used to refine early-stage design development of a microneedle (MN) patch as a new drug delivery system for long-acting, progestin-only contraception for women.

 Researchers assessed perceptions and preferences of users and stakeholders through iterative rounds of focus group discussions and interviews with participants in four countries. After exploring user/stakeholder perceptions from Uganda, The Gambia and the United Kingdom, prototypes were fabricated representing “ideal” product characteristics which were evaluated for acceptability and ease of use in Malawi. Potential users and providers were enthusiastic about the concept of a new type of product that would provide long-term protection (6 month duration), might be more convenient than other hormonal options, and has the potential for self-administration. User experience and perspectives helped refine the applicator design and the feedback mechanism, and informed preferences about packaging and instructions for use.

 This article provides an important contribution to the growing body of work showing that user-centered development processes can be used when developing sexual and reproductive health products and provides an example about how user/stakeholder feedback can inform design decisions.

 Below are some comments/questions/suggestions for the authors to consider which may strengthen comprehension of this article.

 1. Page 3. Methods: Concept Development. Re: “The concept of a new MN-based progestin-only hormonal contraceptive delivered by an applicator was developed by the team (Design Stage 1, Figure 1) after reviewing the available literature and discussing the expectations of stakeholders regarding novel contraception solutions with experts in the field of FP in LMICs.”

- Perhaps share a bit about what were these expectations that helped shape the initial design thinking about this contraceptive MN approach? Would be helpful to know the starting position for this concept so readers can see how this changed/evolved after user/stakeholder inputs. Did the concept of the MN patch change based on user input or simply the design features and characteristics?
- The description of MN states they can deliver drug for therapeutic duration longer than 1 month, but in developers start with the assumption this would be a 6-month duration method. So, it might be helpful to clarity where that assumption comes from and why that is the target (especially since users from multiple countries talk about the desire for a MN patch with shorter duration to confirm it is appropriate for them).

2. The authors propose that adding a new method may help address unmet need for contraception. It is well-documented that adding a new option to the method mix can result in increased uptake of contraception overall, but simply adding another hormonal contraceptive delivery system does not seem to address key reasons identified by The Guttmacher Institute analyses as reasons for unmet need for contraception (see: Unmet Need for Contraception in Developing Countries: Examining Women’s Reasons for Not Using a Method https://www.guttmacher.org/report/unmet-need-for-contraception-in-developing-countries) such as concern about side effects and infrequent sex. So, it might be helpful to more clearly understand where the developers think this product would fit in the contraceptive method mix; what is the gap they are aiming to fill and the value added by this new technology; and who are the potential users that a product like this might be most useful/attractive for.

 3. Page 5 Figure 2. Diagram illustrating the different steps of a Human Centred Design study. “After defining the area of innovation, the target end users and the context of use are considered to ideate possible solutions for the unmet need that is being addressed. The proposed solutions are tested by potential end users and their feedback is incorporated into a refined design. Multiple iterations of this process lead to the development of the final product.”

- While this statement is accurate, this description overlooks the potential importance of input from other stakeholders who influence whether a contraceptive product will be approved, come to market and become integrated into the healthcare system in specific countries. Collecting input from those other stakeholders may be outside the scope of this set of studies, but it should be acknowledged that developing an acceptable product that meets end-users needs and context of use is not sufficient to guarantee that the product will make it to commercialization and introduction/scale up. 
- A key factor not much mentioned in this article relates to cost (manufacturing cost/cost of service delivery/cost to the consumer) relative to other currently available contraceptive products. Design considerations often have underlying cost implications. Cost will be an important consideration for a potential commercialization partner looking at the potential viability of this technology and also for donors, NGOs, procurement agencies, *et al.* involved in contraceptive supply. Perhaps the authors could state that cost considerations (design/manufacturing) were considered, but not reported at this time, or something like that?

Sampling and recruitment:

 4. It might be helpful to add a statement about how/why these countries were selected as representative for user/stakeholder assessments. Given that 3 of the countries are in Africa, it seems the researchers are focusing on unmet need for FP in LMIC. If so, it would be helpful to also understand their rationale for including participants from UK, especially since the UK user groups (user studies 3 and 4) represent a large percent of the overall participants.

 5. The authors identify unmet need for contraception globally as the rationale for developing the MN contraceptive patch. So, it might be helpful to better understand why they recruited such a high percentage of participants who already are using contraception (only 18 of 88 participants reported not currently using contraception; 14 participants did not respond to this question). Was this because persons using contraception were easier to identify and recruit? Or some other reason? Do the researchers feel they may have missed something by not more specifically including viewpoints of those not currently using contraception? Or those who were specifically dissatisfied with their current contraceptive method?

 6. Page 6. It is surprising that the FGDs in Uganda and The Gambia were conducted in English by members of the research team rather than in local language (or at least to give the participants the option of which language to use) and with a local facilitator who is closer in culture/social location to the participants. Does the research team see this as a potential limitation of this study design? Or do they think this did not have any impact on their results and interpretation of findings?

 7. Page 7. Given that the assessments moved sequentially from country to country and that inputs from user studies 1,2,3 were validated by users in studies 4 and the final design in user study 5, it seems there was little opportunity to go back to previous participants and double check that the changes incorporated into the refined prototype design correctly addressed the needs identified and that the developers interpreted the preferences of participants from previous rounds of evaluation correctly. Is this accurate? Given that the profile of potential users in the UK (User Studies 3 and 4) differs from users in Uganda and The Gambia (studies 1 and 2), and we don’t have a complete profile of users from Malawi (study 5), are the researchers confident that user needs have been adequately addressed in the final prototype…or is it possible that something has been lost in translation as they moved to these different user groups.

 8. Page 7. “During FGDs and SSIs with potential users, providers and organisations, dummy prototype MN applicators were used to obtain feedback on handling/ergonomics and aesthetics.” What do you mean by dummy prototype applicators? Were these functional prototypes (but without drug) that gave the look, feel, usability? Did the participant use the applicators in mock use? Is it possible to include an image of what these looked like?

 9. Page 8. The demographic profiles in Fig 4 are not all equally complete. This makes it difficult to compare similarities/differences among user groups across countries. 

- Perhaps add an explanation about why the profile for Malawi does not include age, education, or religion. Also do the researchers have any explanation/insight for why 2/3 of participants in Malawi did not respond to the questions about living situation and whether or not they have children? Without this information it is difficult to tell how similar or different the Malawi group is to the initial user groups in Uganda and The Gambia. This is important because Malawi is used to validate the final design.

- Also, only a small number of potential users (2) fall in the 18-20 age group, yet we know that unmet need for contraception is high among young women, especially young unmarried women. Do the researchers feel this may be a limitation of this study?
- The text says participants representing both urban and rural locations were recruited but we don’t see this in Fig 4. What was the breakout of urban/rural participants? Is it possible to include this?

10. Page 8.  Fig 5. Providers. Given that part of the intended value proposition of MN patch is self-administration, perhaps the authors can clarify why they included so many providers in these assessments (slightly more providers than potential users). In retrospect, do the researchers feel this was appropriate break down of participants?

 11. In the text, the authors mention collecting input from male partners, but I don’t see reference to any findings or male partner input in the text.  Were the interviews not productive? Did the male partners have anything interesting to share that influenced design, or provided insights about potential acceptability, or support or concern for their partner potentially using a product like this?

 12. Pages 8 and beyond. The researchers assessed perspectives and preferences of a range of potential users and stakeholders across multiple countries. Did the researchers find differences between any of the user groups that were relevant or interesting?  (perhaps based on age, education, contraceptive use, relationship status, or urban/rural location?) Were any comments particularly insightful/enlightening or surprising? Were there comments that pointed toward design trade-offs that needed to be made to fit within what was technically feasible or within the constraints of the project? If so, that would be interesting to bring forward. I feel like the voices of the users/stakeholders are a bit buried in the description of the process and the results - maybe perhaps that is because this is a summary of 5 user studies.

 13. It would be helpful if the authors could include images or illustrations to show how the MN patch design and/or applicator evolved based on input from user/stakeholders. If that is not possible, perhaps add a statement indicating why images cannot be included at this time.

 14. Overall, I found it difficult to follow which participants evaluated the MN concept via illustrations compared to those who handled and practiced using the MN prototypes and still am not sure if any of the participants used the prototypes in mock use. Is there a way to help make this more clear?

 15. Page 9. “…the transport/disposal cap was improved to increase childproofing and prevent accidental exposure after disposal." This is a bit confusing. What is the risk of exposure during disposal since there are no needles and the drug has been delivered. Also, the text describes that the disposal cap was improved, but without any illustration or image, it is hard to understand what this means. 

 16. Page 9. “Each illustrated step was also detailed with text, to allow users to correctly apply the product by reading the text and/or looking at the imagery. This improved instruction leaflet was subsequently presented to participants in Malawi (User study 5), who considered it appropriate.” 

- What does “appropriate” mean here? Appropriate in style and format? Did the Malawi participants use the instructions to learn how to use the MN patch in mock use? Or did they just look at the IFU and comment on these? Were the images and text evaluated for comprehension? (FYI. There is a body of literature about creating instructional health materials for low literate audiences. Best practices include combining images and simple messages, so the authors findings in and of themselves are not surprising. Not sure you need to reference this, but here is a guide developed by PATH in 1996 that still contains helpful and relevant guidance as the authors move forward: https://path.azureedge.net/media/documents/DC_Low_Literacy_Guide.pdf).

17. Page 14. Discussion: “ A method that requires minimal training would be more accessible and with this in mind our research team have developed a user informed device that is simple to use and can potentially be self-administered.”

- Since this is intended to be simple to use, it would be helpful to outline the steps or show the IFU, perhaps in the supplemental information?

Is the work clearly and accurately presented and does it cite the current literature?

Partly

If applicable, is the statistical analysis and its interpretation appropriate?

Not applicable

Are all the source data underlying the results available to ensure full reproducibility?

Yes

Is the study design appropriate and is the work technically sound?

Yes

Are the conclusions drawn adequately supported by the results?

Yes

Are sufficient details of methods and analysis provided to allow replication by others?

Yes

Reviewer Expertise:

I am a reproductive health professional with 35 years of experience at PATH. I’ve worked in the areas of contraceptive and reproductive health technologies, product development, regulatory approvals, contraceptive introduction, research, clinical trials, and HIV/AIDS.

I confirm that I have read this submission and believe that I have an appropriate level of expertise to confirm that it is of an acceptable scientific standard, however I have significant reservations, as outlined above.

# Response

**Published:** 2021-11-25

**Authors:** Benedetta Gualeni

**Affiliations:** Cardiff University, UK

We thank the reviewer for their positive comments relating to the important contribution that this article will make. We address specific comments in turn below.

 1. We have reflected on the first bullet-point of this comment and to a similar comment from the other reviewer and have added the following narrative to the manuscript.

 “The initial concept was to develop a long-acting, reversible, MN-based, progestin-only hormonal contraceptive, that is delivered by an applicator, can be self-administered (or administered with minimal training), is stable at high temperatures and high humidity, and can be mass produced at low cost. An assortment of potential products, with different features (Design Stage 1, Figure 1) were therefore developed. These initial product designs were informed by the published literature, the opinions of stakeholders with experience of developing and/or using FP in LMICs and the multidisciplinary research team, which includes product designers, engineers, pharmaceutical scientists and polymer scientists.”

 In response to the second question raised in the first point, elements of the concept were refined or reinforced following user input, e.g. the desired length of time for contraception, and specific features of different products, e.g. the size and the ergonomics, that were identified as attractive were used to inform product design.

 We have reflected on the second bullet-point of this comment, and also on a similar comment from the other reviewer, and we have added the following narrative to the manuscript to better clarify this issue:

 “Injectable contraceptive products provide 12 weeks (3 months) of contraception and implants provide >12 months of contraception to the user. The proposal was therefore to develop a product with an alternative duration of action that would provide users with another contraceptive choice. Based on current technology, a 6-month duration of action is at the technical limit of what can be achieved using a relatively small microneedle array patch, considering drug loading limitations and the preferred size of patch indicated by potential users”.

 2. As stated in the addition we made to the manuscript following the previous comment, injectable contraceptive products provide 12 weeks (3 months) of contraception and implants provide >12 months of contraception to the user. The proposal was therefore to provide an alternative duration of action that would provide users with another contraceptive choice.

 Another aim was to provide a method of contraception for users that was more discreet than current commercially available long-acting contraceptive implants. 

 This is a hormonal contraceptive though and we would envisage comparable side effects to other hormonal methods, although the lower dose in the proposed intradermal MN delivery system may produce fewer or less severe side effects. This could only be evaluated in clinical studies. Therefore the product may provide a choice for women who have experienced side effects from other forms of hormonal contraception.

 Some users also described how their partners spend half the year working abroad and half the year at home. This type of product may appeal to these women.

 The users that this product may be attractive to are therefore:

- women who don’t have easy access to clinics (e.g. rural setting) and for whom frequent visits to the clinic are burdensome or impractical (interfering with work/daily activities). As noted in the cited report from the Guttmacher Institute, women from West and Central Africa do cite difficult access to a source of supply as a reason for non-use;
- women who would benefit from a longer duration contraceptive (IUDs or transdermal implants), but don’t have access to facilities with trained medical staff;
- women who would prefer a more discreet method that could be self-administered;
- women who are returning to fertility after having had a child, who may want more children but would like a longer interval between pregnancies;
- women that are currently unhappy with their contraceptive options

3. These two points are very important, and indeed cost did influence some of our manufacturing decisions. We’ve added the following paragraph to the manuscript in response to this comment.

 “This process however does not guarantee that the final product will be commercially viable or approved in a particular territory, with other issues such as manufacturability, scalability, regulatory issues, cost of goods, market access and distribution also playing important roles. These issues are also being scrutinised in product development, but are not reported here as they are outside of the scope of this article.”

 4. The countries for Stage 1 were carefully chosen to maximize the type of feedback gathered. We wanted to perform the studies in 2 countries in SSA, one in the West and one in the East, one with a Christian and one with a Muslim majority. The aim was to elicit a range of viewpoints considering different unmet needs and different usage of contraception as highlighted by FP202 ( Uganda | Family Planning 2020 ; Gambia | Family Planning 2020 ). The Gambia and Uganda were chosen due to existing academic or charitable links with the 2 countries and confirmed in discussions with the BMGF.

 With the research group based in the UK, it was considered valuable to gain insight from UK users as well, to explore the appetite for a new LARC in an industrialised country, as revenue from industrialised countries might create the opportunity to subsidize access in LMICs.

 Finally, we considered the value of returning to either Uganda or The Gambia for the final, summative study, but we ultimately decided to perform this study in a different SSA country to widen the range of viewpoints further by bringing in a new population. This would allow opportunity for new perspectives, not previously identified, to be explored. Again, due to existing links in the country, the decision was taken to perform these studies in Malawi.

 We have added a short paragraph in the manuscript to clarify this point.

 “Uganda, The Gambia, and Malawi were selected amongst FP2020 countries due to existing academic or charitable links. Studies in the UK were conducted to explore the views on a new long-acting contraceptive in an industrialised country.”

 5. This comment raises some important points worth clarifying. Accessing non-users to provide their views is always challenging, and in this study there were added logistical reasons which made this more difficult. During our limited duration field trips in Africa, logistically the easiest way to organise focus groups with providers and users was to visit FP clinics or hospitals and recruit participants there. A high percentage of the people attending FP clinics are already users of contraception, with only a few attending to enquire about the different options available as their first contraceptive, as a different contraceptive to what they have used in the past (and they haven’t been happy with), or as a method to return to as they stop breastfeeding and return to fertility. Nevertheless some focus groups were organised outside of the clinical setting and included women with different backgrounds and different experience with use/non use of contraception, but recruitment of these individuals was more challenging, especially given  the short period of time available, as women were a bit more wary to engage with us outside of the clinical setting.

 Ideally, with logistics being less of an issue we would have liked to speak with more individuals and we definitely feel that we are missing some viewpoints, especially from potential users of a young age and those living in a more rural setting. That said, the input from healthcare providers that regularly talk to these groups provided their views on these individuals’ preferences, concerns and needs and this gave us confidence that this method might be popular with some of the women who are currently non users.

 6. The health professionals involved in the study all spoke English well and were comfortable conversing in English. For the potential users, some likewise had a very good mastery of the English language, and fellow participants in focus groups were helping each other if they had problems understanding some particular terms. In both countries a local contact was available to provide translation support during focus groups, with whom we held meeting explaining in detail the scientific background of the project beforehand. This was typically a local health professional.

 Two FGDs in Uganda and two  one-to-one interviews in the Gambia did take place predominantly in the local language, with the local facilitator providing translation support both in asking questions and reporting back answers. These were a bit more tricky to conduct, as the flow and rapport were disrupted. In addition, it wasn’t possible for us to understand if the translator was being neutral or was unconsciously guiding the participant’s responses, or changing the participant’s words when translating back into English.

 This was potentially a limitation of the study, and that’s one of the reasons why we included Malawian researchers in the team for the summative study. These trained researchers had experience of qualitative data collection, knowledge about the project, could run focus groups in the local language, thus minimising the impact of bias resulting from language and translation issues.

 Our findings from the studies in Malawi very closely mirror the ones from studies in The Gambia and Uganda, giving us confidence that the language barrier didn’t have a major effect in early studies.

 7. The reviewer is correct in pointing out that there was no opportunity to go back to previous participants to confirm the changes made were reflective of their feedback. Even returning to the same country for further studies wouldn’t have guaranteed the presence of previous participants in the new study. Therefore it is possible that some of the users’ needs haven’t been accurately addressed during further iterations. It is worth noting that the main applicator features that were changed were:

- The addition of auditory and visual feedbacks to confirm correct deployment of the applicator. This was requested in some form in almost all of the FGDs and users in Malawi universally liked these, even if they hadn’t seen a previous version without feedback. We are considering running another study in the UK presenting participants with 2 versions of the applicator: one with and one without auditory and visual feedback to gather their opinion.
- The stiffening of the transport/disposal cap, to make the applicator childproof. Even though we were not able to go back to participants to ask their opinion about this feature, we are convinced that the cap now serves also for this purpose. It’s worth noting that a final packaging solution that will be developed prior to commercialisation might make a transport/disposal childproof cap redundant, so this feature will be re-evaluated in the future, keeping in mind that users will welcome some sort of childproofing feature.
- The increase in the application force to prevent accidental deployment. Even though the users didn’t specify a particular force desired to actuate the applicator, we increased this force so that the applicator will be very difficult to deployed accidentally, but still easy to actuate with one hand during application.
- Efforts have been made to maintain the applicator as small as possible, as per user preferences. Even though users haven’t specified a desired size, since the publication of this study a lot of efforts have gone into reducing the size of the MN product to allow for a smaller applicator size.

We are therefore relatively confident that the main, general points raised during our studies in different country have been addressed. We keep in mind though that prior to commercialisation some other changes might need to be made to overcome some technical issues, and users feedback will be requested again at later stages.

 8. The dummy prototypes consisted of the fully functional applicators without MNs (just a flat plastic surface where the MNs would be). This allowed participants to experience the mechanism of action, usability, duration of application, feedback from the applicator, etc without having any MNs pierce their skin. Participants were shown how to use the applicator and then performed multiple mock application on themselves and on fellow participants to familiarise themselves with the prototypes. Participants were then shown some drug free MNs under a magnifying glass to help them understand the small size and shape of these devices.

 We have added the following paragraph to the manuscript to clarify this issue:

 “The dummy prototypes used in the studies consisted the fully functional applicators without the presence of MNs. This allowed participants to experience the mechanism of deployment, usability, duration of application and feedback from the applicator.”

 Unfortunately, we are not able to share any images of the prototypes at the moment, as publishing them will constitute prior art that will interfere with securing IP for the novel mechanism used with the applicator (see response to point 13 for more details), but we strongly feel that it is in the best interests of the project, the development of the product and the community of researchers working in this space to publish these results now, even if we cannot show the actual prototype employed. This allows others to benefit from the user studies and does not restrict the potential development and commercialisation of the MN product.

 9. Regarding the first bullet-point, we agree that it would be an advantage to have the complete demographic information and that we are particularly lacking in demographic data within the Malawi cohort. Participants were asked to complete a short demographic survey (provided as supplemental material with the paper) but ethically they had the right to leave some questions blank if they were not comfortable sharing that information and this accounts for some gaps in the data. It would not be ethical to not use this data, even if the demographic data were not complete. Whilst we may have placed more emphasis on the importance of collecting information on these demographic questions (while respecting participants’ rights) it was not practical or possible to retrospectively collect this data. It is important however to use the data obtained, rather than discount data from an important group of participants, even without the demographic context. Any future user studies with finalised product design will ensure that the demographic data is more complete.

 Regarding the second bullet-point, we do feel that his is a potential limitation. As stated above (answer to point 5) it was difficult for us in this study to reach young unmarried women, whose views on the proposed product would have been extremely valuable. Input from healthcare providers that regularly talk to these groups gave us confidence that this method might be popular with this group of potential users. It should also be noted that completing this study during the COVID-19 pandemic made it more difficult to ensure that all of the potential users were targeted.

 Regarding the third bullet-point, the text says that potential providers with experience in different work environments (rural or urban) were recruited. Some potential providers who took part in the study reported that they travel regularly to rural locations to provide contraceptives and advice, and are therefore very mindful of the needs of potential users in those settings, which they expressed during our discussions.

 All of the potential users that participated in the study were from an urban environment as, due to logistical factors including the short duration of the field trip, it was not feasible to travel to rural locations to recruit participants, even though we would have liked to. In the text when we refer to recruiting potential users from different living environments we meant, as shown in figure 4, people having different living arrangement, i.e. living with partner only, partner and children, living with children only, living with parents only, or living with parents and children. We acknowledge the terminology used might create confusion, so in the methods section of the paper we have changed the text from “living environment” to “living arrangements”, to keep it consistent with the terminology used in Fig. 4.

 10. This is a very interesting question. Ideally we  wanted a balance between providers and users. The reality was that providers were very excited to talk to us and feed into this study, and everybody wanted to be included in the discussion. As such we ended up with more people wanting to participate than we planned for and we felt it would have been counterproductive to turn some of them away just because we had reached our intended number of participants.

 Conversely, potential users were a bit more cautious and not everybody agreed to be involved in the discussion due to the sensitive nature of the subject. Even though we reached our target number of participants in this group, we found it very challenging to recruit more potential users to match the high number of participants in the providers group. It is important also to note that whilst self-administration will require the user to apply the product, this could be under the guidance of a provider and the providers also have a good understanding of what may be preferred by the user, based on their experience with multiple users.

 11. Thanks to the reviewer for asking this question, which allow us to briefly share the findings from interviewing this group of participants. The interviews with male partners were very interesting, but not especially useful from a design perspective which is the focus of this paper. The few male partners that agreed to talk to us were already supportive of their partners using contraceptives and would welcome a product with fewer side effects and longer duration. They all stressed the importance of spacing children more, in order to give them better education and better prospect for the future, and some of them enquired if a long acting contraceptive for men was also being developed. They had little interest in trying to handle the applicators, hence their lack of input on the design, but they welcomed the concept of a long acting, minimally invasive contraceptive.

 12. One of the most significant differences we found between countries was that users in Malawi seemed to be wary of the painless nature of the applicator, suggesting that they “need to feel pain to know it has worked”, while participants in other countries did not express this view.

 Providers generally had more questions and concerns than participants, and we think this reflects their experience with multiple women with different needs as well as their medical training and scientific backgrounds.

 We found particularly surprising and uplifting that partners of potential users, especially belonging to the younger generations, seemed to be very involved in the FP discussion and welcoming of different methods coming through, also hoping for new male contraceptives to be available in the near future.

 Regarding design trade-offs, the main issue is size. There is a technical limit to how small the patch can be while still delivering 6-months contraception, and the users consistently reported that a small size was preferable, even though they never specified precise dimensions. We are confident that in our product development we are reaching an acceptable patch size, but this will need to be validated in further user studies. Another design issue that wasn’t seen as concern by participants but which we think is important is disposal. Where it was discussed, it was usually just a comment in relation to using usual methods of disposal (e.g. incineration). Neither users nor providers expressed any major concerns about *safe* disposal although, for us, one of the challenges of this project is to design a product that can be safely disposed of at home after use, and work on this matter is still in progress.

 13. As previously mentioned, images cannot be shared at this time due to IP issues. Hopefully we will be able to share them in a future publication focused more on the technical development than the design and usability element.

 In reference to this comment and also comments made by the other reviewer, we have added the following statement to the manuscript:

 “At this stage we are not able to share any images of the prototypes, as publishing them will constitute a prior art disclosure that will inhibit our ability to protect the novel mechanism used within the applicator.”

 14. We apologise if this information was not clearly conveyed and have modified the manuscript accordingly:

 “All of the participants were first shown the visual renderings, to familiarise them with the basic concept. After the concept was explained using these illustrations, participants were encouraged to ask general questions regarding the concept (side effects, duration, return to fertility, etc). After this discussion (usually lasting 15-20 minutes) all participants were shown the dummy prototypes (without MNs) with examples of associated instruction leaflets. All participants then had the opportunity to handle and deploy the prototypes multiple times (on themselves and/or on other participants). After handling the prototypes for about 10 minutes, participants were asked to provide specific feedback on the applicators (ease of use, size, mechanism, aesthetics, etc) for the rest of the discussion (10-15 minutes).”

 15. We apologise that this was not explained clearly. It is possible that a proportion of drug remains in the device after the application, hence the comment on accidental exposure. For IP reasons, as previously explained, we cannot share images of the cap. The improvement made to this cap means that more dexterity and force is required to remove it from the applicator, comparable to child resistant screw-on lids for oral medications, making it more secure.

 16. Thanks for sharing this content. The participants in Malawi were given the mock applicator with instructions and learnt how to perform an application by just following the instructions. They deemed the instruction as “appropriate” as in “suitable for their purpose”, i.e. they were able to learn how to use the applicator by reading the instruction, but they felt that text in the local language would have helped.

 17. Unfortunately we cannot show the IFU without revealing some sensitive IP, so we cannot share it at the moment.
